# CRISPR-Cas Systems in *Bacteroides fragilis*, an Important Pathobiont in the Human Gut Microbiome

**DOI:** 10.3389/fmicb.2017.02234

**Published:** 2017-11-23

**Authors:** Mehrdad Tajkarimi, Hannah M. Wexler

**Affiliations:** ^1^Brentwood Biomedical Research Institute, Los Angeles, CA, United States; ^2^University of California, Los Angeles, Los Angeles, CA, United States; ^3^GLAVA Health Care System, Los Angeles, CA, United States

**Keywords:** *Bacteroides*, virulence, CRISPR-Cas system, phage, immune defense, gut microbiome, pathobiome

## Abstract

**Background:** While CRISPR-Cas systems have been identified in bacteria from a wide variety of ecological niches, there are no studies to describe CRISPR-Cas elements in *Bacteroides* species, the most prevalent anaerobic bacteria in the lower intestinal tract. Microbes of the genus *Bacteroides* make up ~25% of the total gut microbiome. *Bacteroides fragilis* comprises only 2% of the total *Bacteroides* in the gut, yet causes of >70% of *Bacteroides* infections. The factors causing it to transition from benign resident of the gut microbiome to virulent pathogen are not well understood, but a combination of horizontal gene transfer (HGT) of virulence genes and differential transcription of endogenous genes are clearly involved. The CRISPR-Cas system is a multi-functional system described in prokaryotes that may be involved in control both of HGT and of gene regulation.

**Results:** Clustered regularly interspaced short palindromic repeats (CRISPR) elements in all strains of *B. fragilis* (*n* = 109) with publically available genomes were identified. Three different CRISPR-Cas types, corresponding most closely to Type IB, Type IIIB, and Type IIC, were identified. Thirty-five strains had two CRISPR-Cas types, and three strains included all three CRISPR-Cas types in their respective genomes. The *cas1* gene in the Type IIIB system encoded a reverse-transcriptase/Cas1 fusion protein rarely found in prokaryotes. We identified a short CRISPR (3 DR) with no associated *cas* genes present in most of the isolates; these CRISPRs were found immediately upstream of a *hipA/hipB* operon and we speculate that this element may be involved in regulation of this operon related to formation of persister cells during antimicrobial exposure. Also, blood isolates of *B. fragilis* did not have Type IIC CRISPR-Cas systems and had atypical Type IIIB CRISPR-Cas systems that were lacking adjacent *cas* genes.

**Conclusions:** This is the first systematic report of CRISPR-Cas systems in a wide range of *B. fragilis* strains from a variety of sources. There are four apparent CRISPR-Cas systems in *B. fragilis*—three systems have adjacent *cas* genes. Understanding CRISPR/Cas function in *B. fragilis* will elucidate their role in gene expression, DNA repair and ability to survive exposure to antibiotics. Also, based on their unique CRISPR-Cas arrays, their phylogenetic clustering and their virulence potential, we are proposing that blood isolates of *B. fragilis* be viewed a separate subgroup.

## Introduction

Bacteria are constantly exposed to incoming DNA via phages, plasmids, other mobile genetic elements (MGEs) or “naked” nucleic acid from lysed cells. Their ability to incorporate these bits of DNA into their own genetic code, known as horizontal gene transfer (HGT), contributes to the ability of a bacterium to adapt to a wide variety of ecological and environmental pressures, including antibiotics and evolving host niche. Along with this ability to incorporate DNA, bacteria possess immune defense mechanisms to defend themselves against invading DNA or RNA in their immediate environment, including systems to abort infection, specialized enzymes that degrade foreign DNA (restriction/modification or R/M systems), and an adaptive immunity system first described more than a decade ago (Haft et al., 2005) that confers adaptive immunity to invading DNA or RNA (Marraffini, 2015). This system, the CRISPR-Cas system, is defined by clustered regularly interspaced short palindromic repeats (CRISPR) and the CRISPR-associated (Cas) proteins (Haft et al., 2005).

The specificity of the CRISPR system lies in the short, repetitive (sometimes palindromic) direct repeat sequences (DR) separated by nucleic acid sequences (spacers) previously acquired from invading DNA and a cleavage system that can target incoming DNA based on recognition of those previously encountered DNA sequences (Burstein et al., 2016). The Cas protein machinery that mediates the protection against invading nucleic acid is encoded by gene clusters that are adjacent to and generally upstream of the CRISPR locus (Jore et al., 2011). The CRISPR RNA array (crRNA) that includes the entire DR sequence with the intervening spacers is transcribed (from the leader) as a single transcript and later processed into individual segments that contain one spacer sequence and portions of the DR sequence at both ends. These individual crRNAs are eventually used to direct Cas proteins to destroy incoming invasive genetic elements (including phages, plasmids and other nucleic acid segments) that include sequences complementary to one of the spacer sequences in the CRISPR. Newly acquired spacers are incorporated into CRISPR loci in a directional manner, generally, but not invariably, at repeats adjacent to CRISPR leaders (Rath et al., 2015).

Many surveys reported that a vast number of prokaryotic species possess CRISPR arrays (Louwen et al., 2014; including ~45% of bacteria and ~83% of Archaea; Grissa et al., 2007) although there are entire phyla that lack this system (Burstein et al., 2016). CRISPR-Cas systems were classified into 18 structural families and 24 sequence families (Makarova et al., 2015). While new subtypes are still being identified, the major groups include 5 major types and 16 different subtypes. The basis for these classifications include which *cas* genes are present, the *cas* operon architecture, Cas protein sequences, and the nuclease involved in the degradation of the target DNA (Barrangou, 2015). The function of some of the *cas* genes has been described in detail, while the function(s) of other genes remains unknown (Rath et al., 2015). The most common systems described thus far in prokaryotes are variants of the (1) Class 1 Type I system, (2) the Class 1 Type III system, and (3) the Class 2 Type II system (Makarova et al., 2015). In general, most strains of the same species contain identical CRISPR-Cas types (Louwen et al., 2014) although there are some variations.

Since the host CRISPR/Cas can acquire and incorporate incoming DNA as spacer DNA, the resultant CRISPR may serve as a molecular record of past exposure to foreign DNA for an individual strain. Alternatively, entire CRISPR loci may be disseminated via HGT There is evidence that these systems are readily transferred between microbes, potentially even across phylum boundaries (Burstein et al., 2016); this is supported both by the phylogenetic relationship of the *Cas* genes from diverse organisms (Godde and Bickerton, 2006) as well as the fact that DR sequences from diverse organisms can be clustered together into subtypes based on sequence similarities (Kunin et al., 2007).

The function of the CRISPR-Cas system was initially described as protection against invasive nucleic acid. However, the sheer diversity of the systems suggested other functions, and indeed, these elements have now been implicated in regulation of transcription, chromosomal segregation and rearrangement and DNA repair, although most of the processes related to these alternate functions are not completely clear at this point (Stern et al., 2010; Sampson and Weiss, 2014). The potential of regulation via suppressing transcription is also suggested by the broad use of modified Cas9 nuclease precisely to suppress transcription in engineered systems (Barrangou et al., 2007; Sander and Joung, 2014; Mimee et al., 2015; Mougiakos et al., 2016).

The presence of CRISPR-Cas systems are variably associated with virulence (Makarova et al., 2011; Barrangou, 2015) and/or antibiotic resistance in pathogenic bacteria. For example, the presence of a CRISP-Cas locus correlated inversely with acquired antibiotic resistance (Palmer and Gilmore, 2010) in *Enterococcus faecalis*; a mechanism for CRISPR-Cas loss in this species was identified and the data suggested that antibiotic use inadvertently selects for enterococcal strains with compromised genome defense. Conversely, an *E. faecalis* strain harboring CRISPR-Cas was more virulent than one lacking this element (Bourgogne et al., 2008). The type of CRISPR element may also be related to the virulence of the bacterium: CRISPR typing was able to discriminate between lineages of *Propionibacterium acnes* with varying degrees of virulence although there were strains belonging to the more invasive lineages that lacked such spacers or a complete CRISPR-Cas system (Marinelli et al., 2012). On the one hand, the ability of a bacterium to incorporate mobile elements bearing pathogenic determinants would argue for a less robust CRISPR-Cas defense system; on the other hand, the presence of multiple CRISPRs systems with a variety of spacers that may indicate previous exposure to these elements, parts of which were incorporated into the bacterial genome.

While CRISPR-Cas systems were identified in bacteria from a wide variety of ecological niches, there are no studies describing CRISPR-Cas elements in *Bacteroides* species, the most prevalent anaerobic bacteria in the lower intestinal tract. The species *Bacteroides fragilis* (BF), an important gut pathobiont, is of particular interest, since its transition from friendly commensal to dangerous threat is not well understood. Outside its colonic niche, *B. fragilis* is an opportunistic pathogen; *B. fragilis* only accounts for 2% of the total gut *Bacteroides* yet it is the agent of >70% of *Bacteroides* infections. *B. fragilis* is the main cause of anaerobic bacteremia and intraabdominal abscesses, implicated in serious gynecological, soft tissue infections, peritonitis, brain abscess (Wexler, 2014) and surgical site infections (SSIs) following colorectal surgery (Solomkin et al., 2010). Even *B. fragilis* strains isolated as normal gut microbiota possess many genes associated with virulence; as the expression of virulent genes constitutes a form of stress response of pathogenic bacteria during host infection, we would expect differential regulation of the multitude of genes involved in virulence, survival, and host colonization (Louwen et al., 2014).

*Bacteroides fragilis* are also important players in the “hot spot” of HGT between microbes (Kurokawa et al., 2007) and constitute one of the most concentrated reservoirs of resistance genes in the human gut (Salyers et al., 2004). HGT throughout diverse bacterial species has been responsible for the dissemination of both virulence and resistance genes that undermine the usefulness of most antimicrobials (Barlow, 2009). In this gut milieu, resistance genes can move from commensal organisms to potential pathogens, between pathogens, and even between pathogens and probiotics (Capozzi and Spano, 2009). Genetic analysis of *B. fragilis* clinical isolates as well as isolates from the GI tract indicate a high degree of HGT, including resistance genes from very divergent gram-positive bacteria (Husain et al., 2014).

The importance of CRISPR-Cas systems in *B. fragilis* is of particular interest, given the potential of these systems to regulate and/or record HGT, including the acquisition of virulence and antimicrobial resistance genes. CRISPR-Cas systems may also regulate endogenous genes, some of which may be important in the transition of *B. fragilis* from commensal to virulent pathogen. The possible ability of Type I CRISPR-Cas to effect DNA repair would be highly significant for *B. fragilis*, which is very susceptible to the DNA-damaging properties of oxygen exposure. The aim of our study was to investigate and document the occurrence, prevalence and diversity of CRISPR-Cas systems in *B. fragilis* strains with publically available genomes, including strains from a variety of clinical sources and belonging to different phylogenetic clades. With this information, we will explore the capacity of the naturally-occurring CRISPR-Cas systems to regulate gene expression, control HGT (including transmission of antimicrobial resistance and virulence determinants) and control reaction to antibiotic exposure.

## Methods

### Strains

One hundred nine published sequences of *B. fragilis* as well as the complete genome sequence of *B. fragilis* species UW (a blood isolate reported which clusters phylogenetically with a subset of strains from *B. fragilis*; Salipante et al., 2015) were retrieved from the National Center for Biotechnology Information (NCBI, http://www.ncbi.nlm.nih.gov/genome). The assembly numbers of these complete genomes, the common names of the strains, and the type of infection from which they were isolated are listed in Table 1. Some strains were classified as virulent or multidrug resistant without the source of isolation; these are so noted.

**Table 1:** *Bacteroides fragilis* strains used in this study.

**Common name**	**Clinical source**	**GenBank accession no**.	**Assembly number**	**References**
*Bacteroides fragilis NCTC 9343*	Appy Abscess	CR626927.1	ASM2598v1	NCTC Type Strain
*Bacteroides fragilis 638R*	Appy Abscess	FQ312004.1	ASM21083v1	Privitera et al., 1979
*Bacteroides fragilis HMW 615*	Appy Abscess	JH815491.1	Bact_frag_HMW_615_V1	Pumbwe et al., 2007a
*Bacteroides fragilis DCMSKEJBY0001B*	Blood	JMZX02000001.1	DCMSKEJBY0001B2.0	Sydenham et al., 2015
*Bacteroides fragilis DCMOUH0067B*	Blood	JPHS01000001.1	DCMOUH0067B_1.0	Sydenham et al., 2015
*Bacteroides fragilis HMW 610*	Blood	JH815482.1	Bact_frag_HMW_610_V1	Sherwood et al., 2011
*Bacteroides fragilis HMW 616*	Blood	JH815524.1	Bact_frag_HMW_616_V1	Pumbwe et al., 2007a
*Bacteroides fragilis DCMOUH0017B*	Blood	JMZY02000001.1	DCMOUH0017B2.0	Sydenham et al., 2015
*Bacteroides fragilis DCMOUH0018B*	Blood	JMZZ02000001.1	DCMOUH0018B2.0	Sydenham et al., 2015
*Bacteroides fragilis 894_BFRA*	Blood	JUOZ01000315.1	ASM105877v1	Roach et al., 2015
*Bacteroides fragilis YCH46*	Blood	AP006841.1	ASM992v1	Kuwahara et al., 2004
*Bacteroides* sp. *UW*	Blood	JANI01000001.1	ASM78502v1	Kalapila et al., 2013
*Bacteroides fragilis DCMOUH0042B*	Blood	JPGQ01000001.1	DCMOUH0042B1.0	Sydenham et al., 2015
*Bacteroides fragilis DCMOUH0085B*	Blood	JPHP01000001.1	DCMOUH0085B_1.0	Sydenham et al., 2015
*Bacteroides fragilis 885_BFRA*	Blood	JUPJ01000001.1	ASM105875v1	Roach et al., 2015
*Bacteroides fragilis 884_BFRA*	Blood	JUPK01000001.1	ASM105874v1	Roach et al., 2015
*Bacteroides fragilis 3397 T10*	ETBF	JGCP01000001.1	ASM59840v1	Science, 2013
*Bacteroides fragilis 1007-1-F #4*	ETBF	JGDK01000001.1	ASM59854v2	Science, 2013
*Bacteroides fragilis 3397 T14*	ETBF	JGDO01000001.1	ASM59916v2	Science, 2013
*Bacteroides fragilis 1007-1-F #9*	ETBF	JGEC01000001.1	ASM59888v1	Science, 2013
*Bacteroides fragilis 1007-1-F #8*	ETBF	JGCM01000001.1	ASM59826v1	Science, 2013
*Bacteroides fragilis 3774 T13*	ETBF	JGCR01000001.1	ASM59830v1	Science, 2013
*Bacteroides fragilis 3783N1-2*	ETBF	JGCS01000001.1	ASM59832v1	Science, 2013
*Bacteroides fragilis 3976 T7*	ETBF	JGCU01000001.1	ASM59816v1	Science, 2013
*Bacteroides fragilis 3986T(B)10*	ETBF	JGCV01000001.1	ASM59818v2	Science, 2013
*Bacteroides fragilis 3986 N(B)19*	ETBF	JGCW01000001.1	ASM59844v1	Science, 2013
*Bacteroides fragilis 3988T(B)14*	ETBF	JGCY01000001.1	ASM59836v1	Science, 2013
*Bacteroides fragilis 3988 T1*	ETBF	JGCZ01000001.1	ASM59820v1	Science, 2013
*Bacteroides fragilis DS-166*	ETBF	JGDD01000001.1	ASM59824v1	Science, 2013
*Bacteroides fragilis S36L11*	ETBF	JGDJ01000001.1	ASM59912v1	Science, 2013
*Bacteroides fragilis 2-F-2 #4*	ETBF	JGDM01000001.1	ASM59882v1	Science, 2013
*Bacteroides fragilis 3719A10*	ETBF	JGDP01000001.1	ASM59884v1	Science, 2013
*Bacteroides fragilis 3783N1-8*	ETBF	JGDR01000001.1	ASM59860v1	Science, 2013
*Bacteroides fragilis J38-1*	ETBF	JGDV01000001.1	ASM59864v1	Science, 2013
*Bacteroides fragilis S6L3*	ETBF	JGDY01000001.1	ASM59922v1	Science, 2013
*Bacteroides fragilis S6R6*	ETBF	JGDZ01000001.1	ASM59924v1	Science, 2013
*Bacteroides fragilis 1009-4-F #10*	ETBF	JGED01000001.1	ASM59870v1	Science, 2013
*Bacteroides fragilis 1009-4-F #7*	ETBF	JGEE01000001.1	ASM59928v2	Science, 2013
*Bacteroides fragilis 3397 N3*	ETBF	JGEG01000001.1	ASM59892v1	Science, 2013
*Bacteroides fragilis 3986 N(B)22*	ETBF	JGEI01000001.1	ASM59894v1	Science, 2013
*Bacteroides fragilis 3986 T(B)13*	ETBF	JGEJ01000001.1	ASM59896v1	Science, 2013
*Bacteroides fragilis S36L12*	ETBF	JGEP01000001.1	ASM59934v1	Science, 2013
*Bacteroides fragilis S36L5*	ETBF	JGEQ01000001.1	ASM59902v1	Science, 2013
*Bacteroides fragilis S38L3*	ETBF	JGEV01000001.1	ASM59876v2	Science, 2013
*Bacteroides fragilis I1345*	ETBF	JGEW01000001.1	ASM59878v2	Science, 2013
*Bacteroides fragilis 3986 N3*	ETBF	JGVG01000001.1	ASM60111v1	Science, 2013
*Bacteroides fragilis 3725 D9*	ETBF	JNHH01000001.1	ASM69968v1	Science, 2013
*Bacteroides fragilis 2-F-2 #5*	ETBF	JGCN01000001.1	ASM59828v1	Science, 2013
*Bacteroides fragilis 34-F-2 #13*	ETBF	JGCQ01000001.1	ASM59842v1	Science, 2013
*Bacteroides fragilis 3783N2-1*	ETBF	JGCT01000001.1	ASM59834v1	Science, 2013
*Bacteroides fragilis 3986 T(B)9*	ETBF	JGCX01000001.1	ASM59846v1	Science, 2013
*Bacteroides fragilis 3996 N(B)6*	ETBF	JGDA01000001.1	ASM59822v1	Science, 2013
*Bacteroides fragilis 3998T(B)3*	ETBF	JGDB01000001.1	ASM59848v1	Science, 2013
*Bacteroides fragilis 3998 T(B)4*	ETBF	JGDC01000001.1	ASM59838v1	Science, 2013
*Bacteroides fragilis DS-208*	ETBF	JGDE01000001.1	ASM59850v1	Science, 2013
*Bacteroides fragilis DS-71*	ETBF	JGDF01000001.1	ASM59908v1	Science, 2013
*Bacteroides fragilis Ds-233*	ETBF	JGDG01000001.1	ASM59880v1	Science, 2013
*Bacteroides fragilis J-143-4*	ETBF	JGDH01000001.1	ASM59852v1	Science, 2013
*Bacteroides fragilis S13 L11*	ETBF	JGDI01000001.1	ASM59910v1	Science, 2013
*Bacteroides fragilis 1007-1-F #7*	ETBF	JGDL01000001.1	ASM59914v2	Science, 2013
*Bacteroides fragilis 3397 N2*	ETBF	JGDN01000001.1	ASM59856v1	Science, 2013
*Bacteroides fragilis 3725 D9(v)*	ETBF	JGDQ01000001.1	ASM59858v1	Science, 2013
*Bacteroides fragilis 3976 T8*	ETBF	JGDS01000001.1	ASM59918v2	Science, 2013
*Bacteroides fragilis 3-F-2*	ETBF	JGDT01000001.1	ASM59886v1	Science, 2013
*Bacteroides fragilis B1 (UDC16-1)*	ETBF	JGDU01000001.1	ASM59862v1	Science, 2013
*Bacteroides fragilis Korea 419*	ETBF	JGDW01000001.1	ASM59920v1	Science, 2013
*Bacteroides fragilis S23 R14*	ETBF	JGDX01000001.1	ASM59866v1	Science, 2013
*Bacteroides fragilis 1007-1-F #10*	ETBF	JGEA01000001.1	ASM59868v2	Science, 2013
*Bacteroides fragilis 1007-1-F #3*	ETBF	JGEB01000001.1	ASM59926v1	Science, 2013
*Bacteroides fragilis 20793-3*	ETBF	JGEF01000001.1	ASM59890v1	Science, 2013
*Bacteroides fragilis 3719 T6*	ETBF	JGEH01000001.1	ASM59872v1	Science, 2013
*Bacteroides fragilis A7 UDC12-2*	ETBF	JGEK01000001.1	ASM59898v1	Science, 2013
*Bacteroides fragilis S23L24*	ETBF	JGEL01000001.1	ASM59930v1	Science, 2013
*Bacteroides fragilis S24L15*	ETBF	JGEM01000001.1	ASM59900v1	Science, 2013
*Bacteroides fragilis S24L26*	ETBF	JGEN01000001.1	ASM59874v1	Science, 2013
*Bacteroides fragilis S24L34*	ETBF	JGEO01000001.1	ASM59932v1	Science, 2013
*Bacteroides fragilis S38L5*	ETBF	JGER01000001.1	ASM59936v1	Science, 2013
*Bacteroides fragilis S6L8*	ETBF	JGES01000001.1	ASM59938v1	Science, 2013
*Bacteroides fragilis S6R5*	ETBF	JGET01000001.1	ASM59904v1	Science, 2013
*Bacteroides fragilis 3783N1-6*	ETBF	JGEU01000001.1	ASM59906v2	Science, 2013
*Bacteroides fragilis S6L5*	ETBF	JGVC01000001.1	ASM60101v1	Science, 2013
*Bacteroides fragilis 1007-1-F #5*	ETBF	JGVD01000001.1	ASM60103v1	Science, 2013
*Bacteroides fragilis S6R8*	ETBF	JGVE01000001.1	ASM60107v2	Science, 2013
*Bacteroides fragilis 1007-1-F #6*	ETBF	JGVF01000001.1	ASM60109v1	Science, 2013
*Bacteroides fragilis S23L17*	ETBF	JHEF01000001.1	ASM60105v1	Science, 2013
*Bacteroides fragilis 86-5443-2-2*	ETBF	LIDS01000001.1	ASM169988v1	Pierce and Bernstein, 2016
*Bacteroides fragilis 20656-2-1*	ETBF	CM004522.1	ASM169987v1	Pierce and Bernstein, 2016
*Bacteroides fragilis 2-078382-3*	ETBF	CM004523.1	ASM169986v1	Pierce and Bernstein, 2016
*Bacteroides fragilis BOB25*	ETBF	CP011073.1	ASM96578v1	Nikitina et al., 2015
*Bacteroides fragilis 2-F-2 #7*	ETBR	JGCO01000001.1	ASM59814v1	Science, 2013
*Bacteroides fragilis KLE1758*	Feces	KQ971009.1	ASM158009v1	HMP^a^
*Bacteroides fragilis CL03T12C07*	Feces	JH724181.1	Bact_frag_CL03T12C07_V1	HMP
*Bacteroides fragilis CL05T12C13*	Feces	JH724193.1	Bact_frag_CL05T12C13_V1	HMP
*Bacteroides fragilis JIM10*	Feces	CM004507.1	ASM169269v1	Russia^b^
*Bacteroides fragilis CL07T12C05*	HMP	JH724215.1	Bact_frag_CL07T12C05_V1	HMP
*Bacteroides fragilis 3_1_12*	HMP	EQ973245.1	ASM15701v1	HMP
*Bacteroides fragilis CL03T00C08*	HMP	JH724173.1	Bact_frag_CL03T00C08_V1	HMP
*Bacteroides fragilis CL05T00C42*	HMP	JH724188.1	Bact_frag_CL05T00C42_V1	HMP
*Bacteroides fragilis CL07T00C01*	HMP	JH724206.1	Bact_frag_CL07T00C01_V1	HMP
*Bacteroides fragilis JCM 11017*	Japan	BAIY01000098.1	ASM61342v1	NBRP, Japan^c^
*Bacteroides fragilis 4g8B_assembly*,	Undernourished Malawian Children	CDQP01000001.1	4g8B_assembly	Kau et al., 2015
*Bacteroides fragilis 2d2A_assembly*	Undernourished Malawian Children	CDQM01000001.1	2d2A_assembly	Kau et al., 2015
*Bacteroides fragilis BF8*	ViR/MDR	LGTH01000001.1	ASM169535v1	Soki et al., 2016
*Bacteroides fragilis S14*	ViR/MDR	CP012706.1	ASM168221v1	Soki^d^
*Bacteroides fragilis 322_BFRA*	Fluid	JVLP01000024.1	ASM105489v1	Roach et al., 2015
*Bacteroides fragilis 321_BFRA*	Fluid	JVLQ01000008.1	ASM105633v1	Roach et al., 2015
*Bacteroides fragilis 320_BFRA*	Fluid	JVLR01000007.1	ASM105486v1	Roach et al., 2015
*Bacteroides fragilis O:21*	ViR/MDR	KV751174.1	ASM169369v1	Soki et al., 2016
*Bacteroides fragilis BFBE1.1*	ViR/MDR	LN877293.1	BFBE1.1	Risse et al., 2015

a *Reference Genome for the Human Microbiome Project (HMP)*.

b *Federal Research and Clinical Center of Physical-Chemical Medicine of FMBA of Russia*.

c *National Bioresearch Project, Japan*.

d *Soki et al. (unpublished), Comparative analysis of Division I and II Bacteroides fragilis strain genomes*.

### Phylogenetic analysis of *B. fragilis* strains

The J species Web Server (with the kind assistance of Dr. Michael Richter, Ribocon, Bremen, Germany) was used to construct a matrix of average nucleotide identity between two organisms (ANIb) values among the *B. fragilis* strains tested. The matrix was converted to a three column array using an Excel macro and then converted into a Newick tree file using a routine in R (with the kind assistance of Dr. Luis M. Rodriguez, Kostas Lab, Georgia Institute of Technology, Atlanta, GA). The Newick file was visualized in the Archaeopteryx software tool (Han and Zmasek, 2009).

### Identification of CRISPRs in *B. fragilis*

CRISPR arrays were identified using both the CRT tool (Bland et al., 2007) with default parameters and the CRISPR Repeat Finder program (Grissa et al., 2007). We found the CRT program to be generally more robust than CRISPR Finder in detecting CRISPRs; on the other hand, CRT does not have a filter to limit acceptable spacer homology within a given CRISPRs, leading to incorrectly identifying tandem repeat sequences (perfect and imperfect) as CRISPRs. The CRT program was used with default parameters and the CRISPR Repeat Finder program was used with the following parameters: Min Number of Repeats 2, Min Repeat Length 19, Max Repeat Length 55, Search Window 8, Min Spacer length 18 and Max Spacer Length 55. CRISPRS repeats that had three or more DR sequences were retained for further analysis and then validated using the CRISPTionary program (Grissa et al., 2007).

### Distribution of spacers among CRISPRs

The CRISPtionary tool (Grissa et al., 2007) was used to analyze spacer distribution and position with individual CRISPRs and to construct a binary file of spacer distribution among the various CRISPR-Cas systems.

### Identification of protospacer target regions

Clustered regularly interspaced short palindromic repeats (CRISPR) targets were identified by submitting the spacer sequences to analysis with the CRISPR Target tool (http://bioanalysis.otago.ac.nz/CRISPRTarget) searching the GenBank Phage, RefSeq Bacteria, RefSeq plasmid and RefSeq viral databases. We also searched the Nucleotide collection (nr/nt) at NCBI using the default parameters, both with and without the limit of “NOT Repeat_Region” added as a query filter. Each matched protospacer was then subjected to a BLAST search (Benson et al., 2014) to find full or partial matches to any other phage, bacteria, plasmid and viruses; annotation of the ten up- and downstream genes flanking the proposed match were recorded.

### Identification of *cas* genes associated with particular CRISPR repeats

Genes flanking specific CRISPR loci were identified by BLAST analysis of the entire CRISPR sequence against the host genome using RAST-based annotation (Aziz et al., 2008). We have found that the RAST annotation server frequently offered a more robust annotation than GenBank and in this study it afforded an easier way to localize the CRISPR element and to view the adjacent genes. Therefore, all bacterial genomes were uploaded to the RAST server and the adjacent *cas* genes as well as the genomic context of the particular CRISPR repeat array were determined. In some cases, the CRISPR was at the end of a gene scaffold, and the upstream genes could not be identified. The particular *cas* operon identified was assigned to a CRISPR-Cas classification system developed by Makarova (Makarova et al., 2011). Most of the *B. fragilis* genome sequences have not been completely assembled, and sometimes the CRISPR repeat sequence was located near the edge of a contig; in those cases, the adjacent genes could not be identified. In those cases, the presence or absence of *cas* genes somewhere else on the genome was noted. Automatic assembly of contigs for genome assembly is often complicated by the presence of transposase genes, and indeed, we found transposase genes adjacent to the CRISPR repeat element in many instances.

### Other bioinformatic tools used in the analysis of *B. fragilis* CRISPRs

Consensus sequences and images for the DR sequences were obtained using WEBLOGO (Crooks et al., 2004). Predicted structures of the DR repeat sequence were visualized using the RNA fold program (Ding et al., 2005). Phylogenetic relationships of the consensus DR sequences in the DR database were analyzed using CRISPRmap (Lange et al., 2013). Gene neighborhoods were visualized using tools at the Joint Genome Institute (Markowitz et al., 2012). Venn diagrams were generated using http://bioinformatics.psb.ugent.be/webtools/Venn/.

## Results

### All *B. fragilis* strains with publically available genome sequences were analyzed for CRISPR repeat arrays

Deep sequence analysis of the CRISPR system was performed for all of the publicly available genome sequences of *B. fragilis* (*n* = 109) (Table 1). The proportion of “pathogenic” vs. “commensal” strains of *B. fragilis* is artificially inflated, since strains from serious infections are more likely to be chosen for whole genome sequencing. A large proportion of these strains are from studies of enterotoxigenic *B. fragilis* (ETBF), so these strains were disproportionately represented and consequently analyzed. Conversely, only a few strains from “normal” feces or human microbiome projects (where samples are often from healthy patients) have been sequenced.

The *B. fragilis* strains included in our study include several blood isolates from geographically distant locations including Washington State (Schapiro et al., 2004), Seattle (Kalapila et al., 2013), Denmark (Ank et al., 2015), the United Kingdom (Pumbwe et al., 2007b), and Afghanistan (Sherwood et al., 2011). A previous phylogenetic analysis of several clinical *B. fragilis* isolates indicated that there is a cluster of *B. fragilis* strains that constitute a genomospecies distinct from other *B. fragilis* (Salipante et al., 2015). Two of these isolates, BF HMW 610 and BF HMW 616, were reported and characterized extensively in our laboratory (Pumbwe et al., 2007a,b; Sherwood et al., 2011). The isolates noted as virulent and multidrug resistant (VIR/MDR) include an international cluster of MDR *B. fragilis* isolates from five countries (Soki et al., 2016). Whole genome sequencing of a large number of ETBF isolates has been undertaken and these are also included (Science, 2013). References, when available, for all of the isolates analyzed are listed in Table 1, and include more details about the origin and nature of these isolates.

### Phylogenetic grouping of *B. fragilis* strains

**Figure 1:**
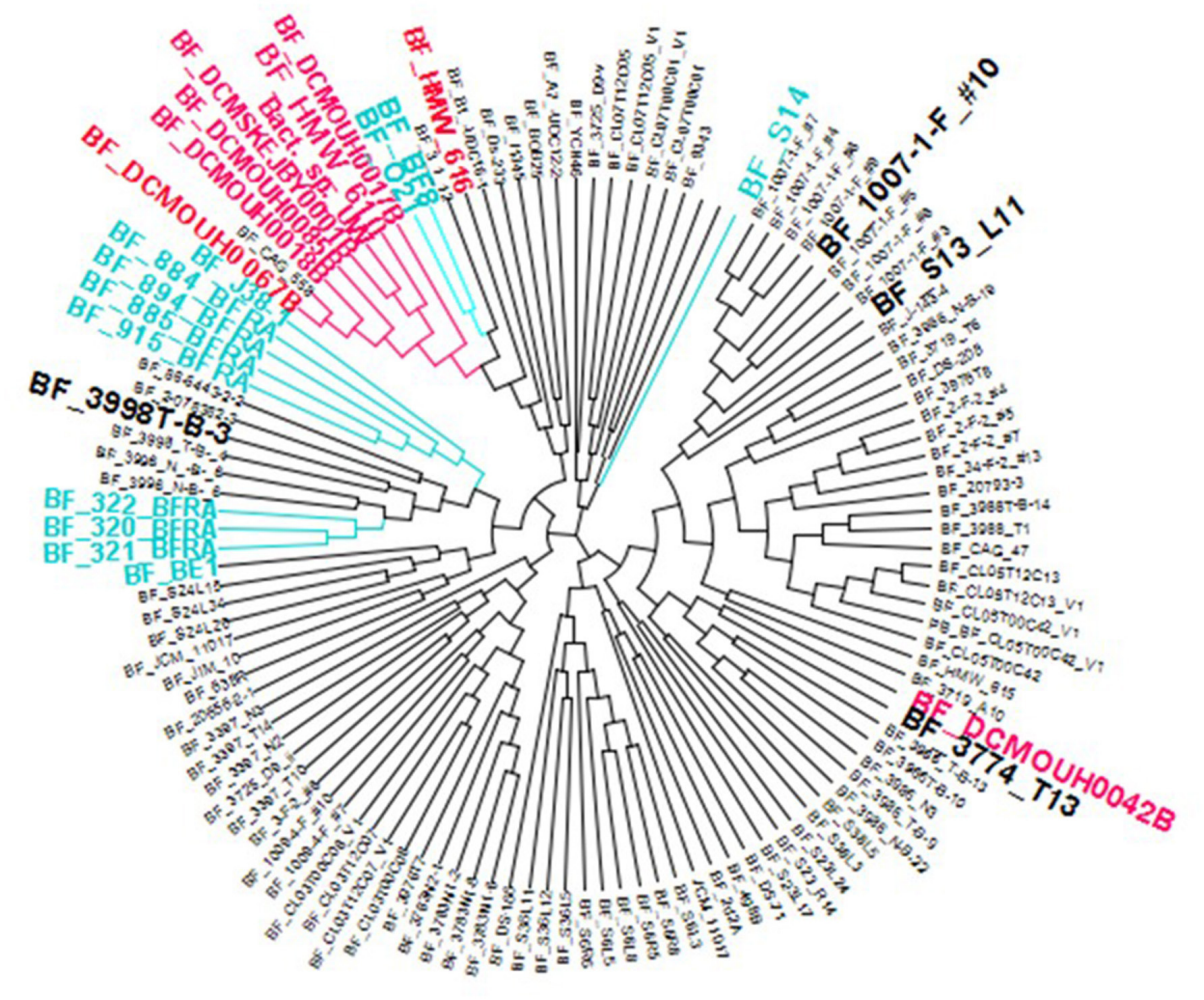
Dendrogram of *B. fragilis* isolates. The dendrogram is based on ANIb values generated for published *B. fragilis* genomes. It is viewed using the Archaeopteryx software tool. *B. fragilis* strains isolated from blood are colored in red. (Although BF Cag 558 is clustered with the blood isolates, there is no available source data). All blood isolates (in red) are clustered together, apart from BF_DCMOUHOO42B (this isolate also has a CRISPR pattern distinct from the other isolates.) Isolates described as multidrug resistant or virulent (teal) are more widely scattered. ETBF isolates are scattered across the dendrogram. Several of the strains are emphasized in a larger black font because their spacer distribution was of particular interest (discussed in Figure 7).

A dendrogram based on Average Nucleotide Identity by BLAST (ANIb) values is shown in Figure 1. All but one of the strains isolated from blood cluster together in one group (despite being isolated in geographically distant areas.) Strains associated with enterotoxin related illnesses formed the majority of the strains and were evenly distributed across the tree as were “commensal” strains isolated as part of the human microbiome project.

### CRISPR-Cas systems in *B. fragilis*

**Figure 2:**
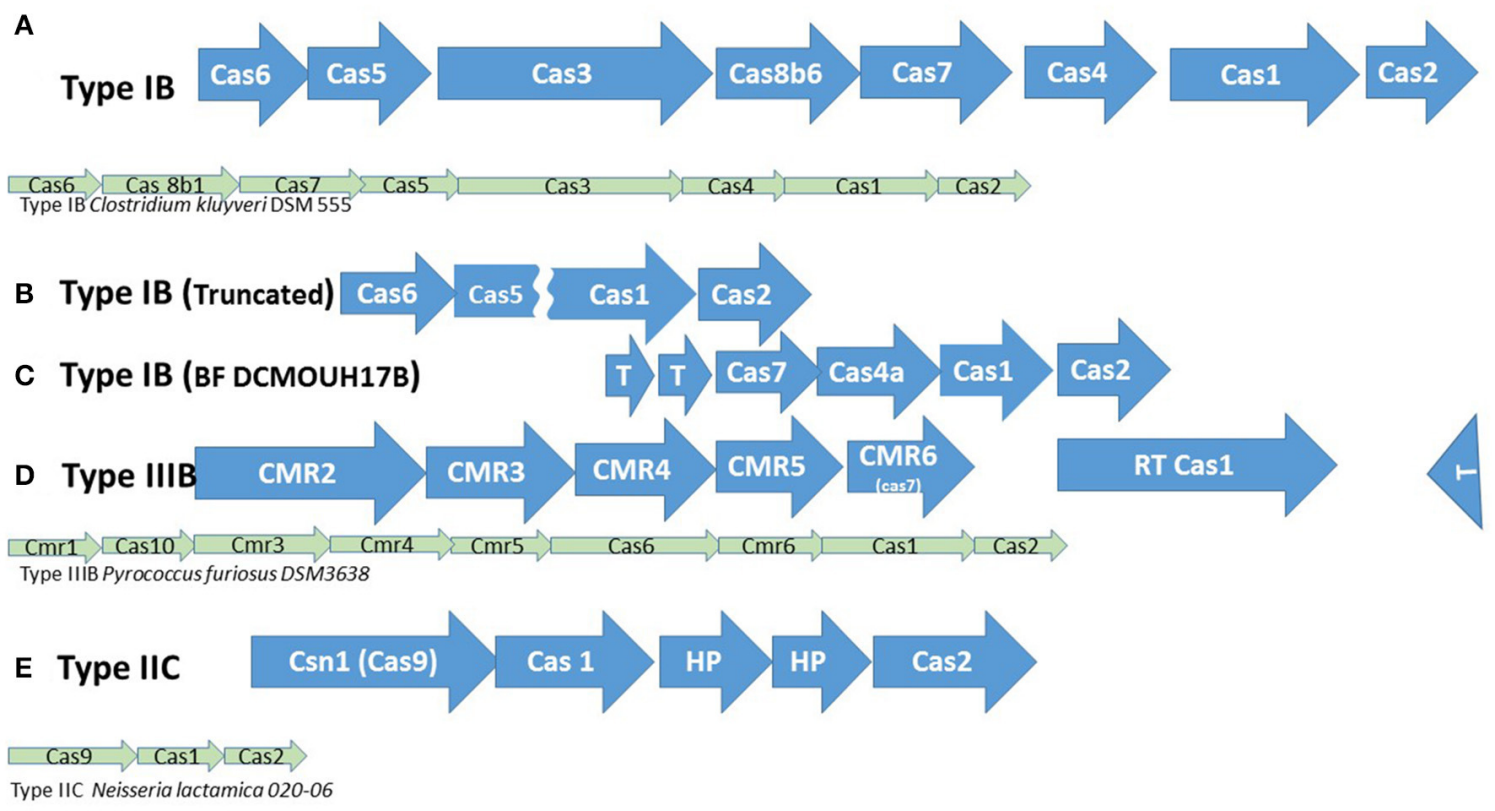
CRISPR-Cas systems in *B. fragilis*. Cas genes found in the respective systems are listed. The canonic arrangement of the closest matching type according to Makarova et al. (2015) is represented by the smaller green arrows below the *B. fragilis* cas operon. *T*, Transposase gene. **(A) Type IB**: The traditional annotation servers identified genes coding for *cas2, cas1, cas3, cas4a*, and *cas6* (TM1814). Three additional genes, classified by all the publicly available annotation sites as hypothetical proteins, have been classified as *cas5, cas7*, and *cas8b6* genes by Makarova et al. (2015); their sequences are very divergent. **(B) Type IB**, **truncated:** These truncated CRISPR-Cas systems were located in the same neighborhood as the complete Type IB CRISPR-Cas systems. The *cas* genes include: *cas2*, truncated *cas1* (252 vs. 1014 bp), truncated *cas5* (381 vs. 564 bp) and *cas6*. The *cas4, cas7, cas8b6*, and *cas3* genes were missing in those strains, as were the 5′ and 3′ ends of *cas1* and *cas5*, respectively. They occur in strains BF 1009 4 F 10, BF 1009 4 F 7, BF 3 F 2-6, BF 3998T B 3, BF KLE1758, BF Korea 419, BF NCTC 9343, BF S14, BF S38L5 and BF s38L3. **(C) Type IB, BF DCMOUH17B:** This array has only some of the genes in the typical Type IB systems. **(D) Type IIIB:** The BF *cas1* gene in the Type IIIB system codes for a reverse transcriptase-Cas1 fusion protein. The other genes present, *cmr2-6* are typical of Type IIIB CRISPR-Cas systems, although in the canonic operon, the order is somewhat different. **(E) Type IIC:** The BF Type IIC system includes the canonic *cas2, cas1* and *csn1* (*cas9*) genes. Two additional genes coding for hypothetical proteins of unknown function are situated between *cas2* and *cas1*.

**Figure 3:**
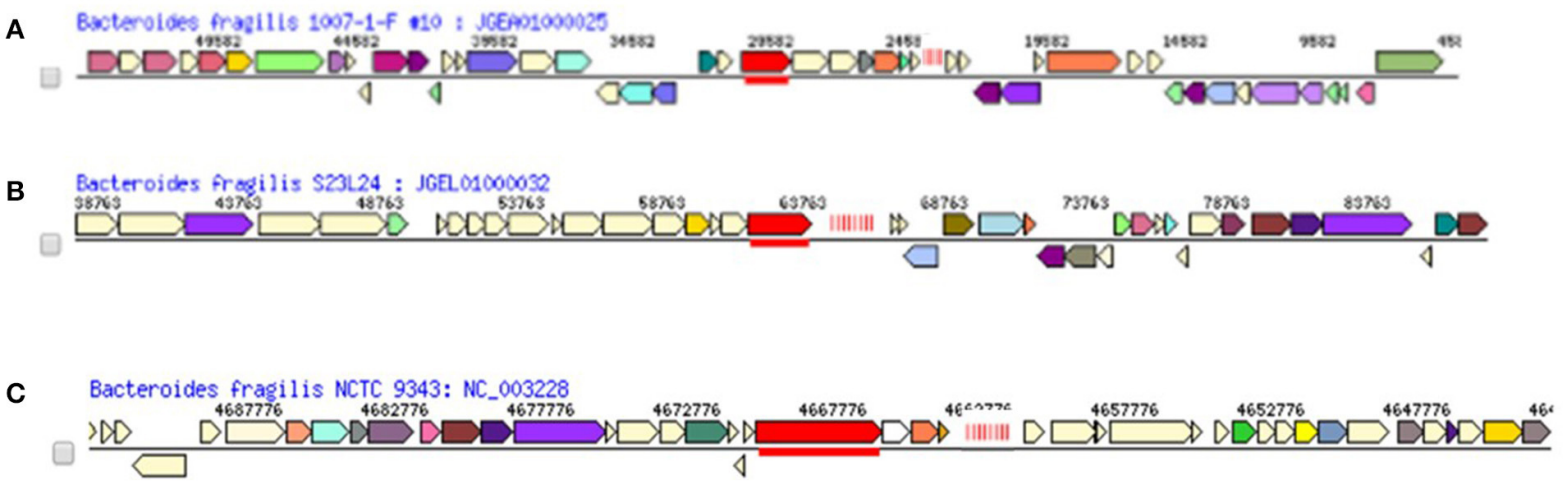
Conserved gene neighborhoods of *B. fragilis* CRISPR-Cas arrays. Proteins coded for by genes are listed below. Vertical red lines represent repeat array. Associated *cas* genes products are in bold. Representative strain is shown with genes surrounding CRISPR-Cas array. The neighborhoods are highly conserved within each CRISPR-Cas type. The most characteristic cas gene for each group (i.e., A: *cas 3*, B: *RT-cas* 1-fusion and C: *cas 9*) are underlined. **(A)** Conserved gene neighborhood of Type IB CRISPR-Cas system in *B. fragilis*. **Upstream genes:** Hypothetical protein;hypothetical protein;Manganese transport protein MntH;Exodeoxyribonuclease III; hypothetical protein; hypothetical protein; hypothetical protein;Translation elongation factor LepA;putative Na+/H+ exchange protein;Na+/H+ antiporter NhaA type;hypothetical protein;DNA recombination protein RmuC; Methionine aminopeptidase**; CRISPR-associated protein, TM1814 family (*cas 6*); hypothetical protein (*cas 5*);CRISPR-associated helicase *cas 3* (underlined); hypothetical protein; hypothetical protein;CRISPR-associated RecB family exonuclease *cas 4a*;CRISPR-associated protein *cas 1*;CRISPR-associated protein *cas 2*; CRISPR REPEAT ARRAY; Downstream genes: Hypothetical** protein;hypothetical protein;RNA pseudouridylate synthase BT0642; RNA methyltransferase, TrmA family;hypothetical protein;Pyruvate,phosphate dikinase;hypothetical protein; hypothetical protein;Thiamin-phosphate pyrophosphorylase;Sulfur carrier protein adenylyltransferase ThiF;Thiazole biosynthesis protein ThiH; hypothetical protein;Thiamin biosynthesis protein ThiC;Thiazole biosynthesis protein ThiG;Thiamin-phosphate pyrophosphorylase; Sulfur carrier protein ThiS; Superoxide dismutase [Fe];ATP-dependent DNA helicase UvrD/PcrA;Carboxynorspermidine decarboxylase, putative;hypothetical protein; **(B)** Conserved gene Neighborhood of Type IIIB CRISPR-Cas system in *B. fragilis*. **Upstream genes:** Two-component system sensor histidine kinase; Two-component system response regulator; Outer membrane protein assembly factor YaeT precursor; ABC transporter permease; Probable ABC transporter permease; putative ABC transporter permease; putative ABC transporter permease; ABC transporter, permease protein; ABC transporter, permease protein; ABC transporter, permease protein; ABC transporter ATP-binding protein YvcR; Thiol:disulfide interchange protein; *M. jannaschii* predicted coding region MJ0978; protein of unknown function DUF88; hypothetical protein; hypothetical protein; hypothetical protein; hypothetical protein; **CRISPR-associated RAMP Cmr2; CRISPR-associated RAMP Cmr3; CRISPR-associated RAMP Cmr4; CRISPR-associated RAMP Cmr5; CRISPR-associated RAMP Cmr6; Retron-type RNA-directed DNA polymerase RT-Cas1 fusion protein (underlined). CRISPR Repeat Array. Downstream genes:** ISNCY family transposase; ISNCY family transposase; Aminotransferase class II, serine palmitoyltransferase like; Transcription regulator [contains diacylglycerol kinase catalytic domain]; Aspartyl-tRNA synthetase; hypothetical protein; N-carbamoylputrescine amidase/Aliphatic amidase AmiE Agmatine deiminase;Ferredoxin domain containing protein; YbbL ABC transporter ATP-binding protein; YbbM seven transmembrane helix protein; hypothetical protein; Possible glyoxylase family protein (Lactoylglutathione lyase); hypothetical protein; Hypothetical protein YbgI; Hypothetical protein; RND efflux system, outer membrane lipoprotein CmeC; RND efflux system, membrane fusion protein CmeA; RND efflux system, inner membrane transporter CmeB. **In contrast, Gene Neighborhood of TYPE IIIB CRISPR Repeat Array in Blood Isolates (absence of *cas* genes) (not shown): Upstream Genes:** Lipopolysaccharide biosynthesis protein; hypothetical protein;UDP-N-acetylglucosamine 2-epimerase; glycosyl transferase, group 1 family protein; hypothetical protein; mannosyltransferase B; UDP-N-acetylglucosamine 4,6-dehydratase; UDP-N-acetylglucosamine 2-epimerase; dTDP-4-dehydrorhamnose reductase; Glycosyl transferase, group 1 precursor; UDP-glucose 4-epimerase; Undecaprenyl-phosphate N-acetylglucosaminyl 1-phosphate transferase; TYPE IIIB REPEAT ARRAY in BF BLOOD ISOLATES; Downstream genes: Putative non-specific DNA-binding protein; hypothetical protein; Na+/H+-dicarboxylate symporter; 6-phosphogluconate dehydrogenase, decarboxylating; Glucose-6-phosphate 1-dehydrogenase; 6-phosphogluconolactonase, eukaryotic type; hypothetical protein; **(C) Gene Neighborhood of Type IIC CRISPR-Cas system in *B. fragilis***. **Upstream gene neighborhood:** Conserved hypothetical protein;putative transmembrane protein;putative transmembrane DNA mismatch repair-like protein;conserved hypothetical protein;putative urocanate hydratase;putative formimidoyltransferase-cyclodeaminase;putative imidazolonepropionase;putative formiminotransferase-cyclodeaminase;putative histidine ammonia-lyase;putative TetR transcriptional regulator;putative outer membrane efflux protein;putative membrane fusion protein transporter;putative transmembrane Acr-type transport protein;conserved hypothetical protein;putative transmembrane protein;conserved hypothetical protein;putative transmembrane polysaccharide modification protein;hypothetical protein;hypothetical protein;hypothetical protein; ***csn1(cas9)*** **(underlined);conserved hypothetical protein (pseudogene);*****cas1;cas2;*** **CRISPR REPEAT ARRAY. Downstream:** Putative transmembrane protein;conserved hypothetical protein;putative transmembrane MotA/TolQ/ExbB proton channel family protein;conserved hypothetical protein;conserved hypothetical protein;putative TonB-family outer membrane receptor protein;conserved hypothetical protein;putative TPR-repeat family protein;putative ATP-binding component of ABC transporter;putative transmembrane protein;putative GntR family transcriptional regulator;conserved hypothetical protein;conserved hypothetical protein;putative ABC transporter transmembrane component.

**Figure 4:**
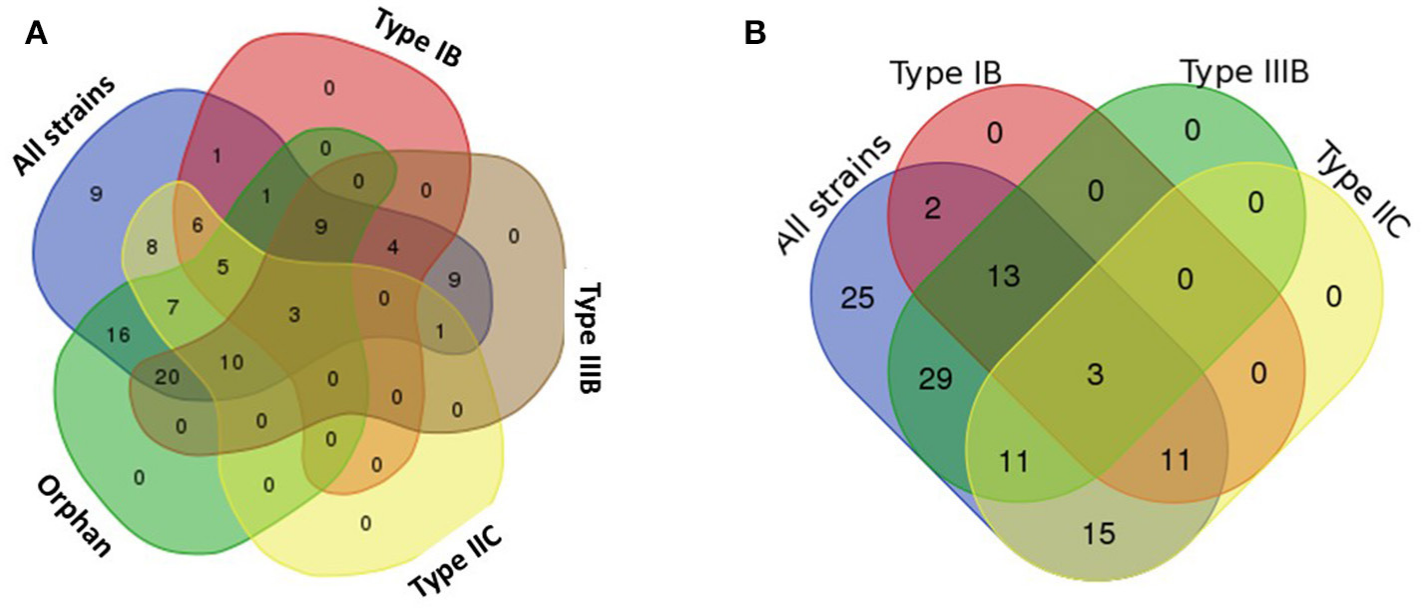
Venn diagram of distribution of CRISPR-Cas systems among strains of *B. fragilis*. The Venn diagrams in Figure 4 include CRISPR-Cas repeats of a given type, whether full or truncated; whether or not there was a full set of adjacent *cas* genes is detailed in Table 2. **(A) Distribution of all CRISPR arrays in *B. fragilis*. Orphan Type IB Type IIC Type IIIB-3 Strains:** BF S38L3, BF S38L5, BF S14. Orphan Type IB Type IIIB-9 Strains: BF 1007 1 F 4, BF 1007 1 F 3, BF Korea 419, BF 1007 1 F 6, BF 1007 1 F 7, BF 1007 1 F 9, BF 1007 1 F 8, BF 1007 1 F 10, BF 1007 1 F 5. **Orphan Type IB Type IIC-5 Strains:** BF 1009 4 F 7, BF KLE1758, BF 3-F-2 #6, BF NCTC 9343, BF 1009 4 F 10. **Orphan Type IIC Type IIIB-10 Strains:** BF 3783N1 8, BF 3783N2 1, BF 3976T7, BF DS 71, BF S23L17, BF S23L24, BF S23 R14, BF 3783N1 6, BF DCMOUH0042B, BF 3774 T13. **Type IB Type IIIB-4 Strains:** BF DCMSKEJBY0001B, BF S13 L11, BF HMW 610 Bacteroides sp. UW. **Type IB Type IIC-6 Strains**: BF 3996 N B 6, BF 321, BF 322, BF 3998T B 3, BF 320, BF 3998 T B 4. **Orphan Type IB-1 Strain**: BF YCH46. **Type IIC Type IIIB-1 Strain:** BF 3783N1 2. **Orphan Type IIIB-20 Strains:** BF 3986T B 10, BF DCMOUH0018B, BF S6L8, BF S36L12, BF 3986 T B 9, BF S6L3, BF DS 166, BF 3988T B 14, BF S6R8, BF S6L5, BF S36L5, BF 3986 N B 22, BF 3988 T1, BF S6R6, BF 3397 N2, BF S36L11, BF 3397 T14, BF 3986 T B 13, BF S6R5, BF 3986 N3. **Orphan Type IIC-7 Strains**: BF 34 F 2 13, BF 2 F 2 4, BF 2 F 2 5, BF S24L26, BF S24L34, BF S24L15, BF BE1 1. Type IB-1 Strains: BF DCMOUH0017B. **Type IIIB-9 Strains**: BF 3397 T10, BF 3976 T8, BF DCMOUH0067B, BF J38 1, BF 894, BF 885, BF 884, BF 3719A10, BF 3397 N3. **Type IIC-8 Strains:** BF 3719 T6, BF 3725 D9 ii, BF 2 078382 3, BF 20656 2 1, BF DS 208, BF 3986 N B 19, BF 638R, BF 86 5443 2 2. **Orphan-16 Strains:** BF BOB25, BF A7 UDC12 2, BF CL07T00C01, BF CL03T00C08, BF HMW 616, BF 2 F 2 7, BF 3 1 12, BF CL07T12C05, BF CL03T12C07, BF JCM 11017, BF JIM10, BF, BF8, BF I1345, BF 20793 3, BF B1 UDC16 1, BF O: 21. **No CRISPR-Cas- 9 Strains:** BF CL05T00C42, BF HMW 615, BF DCMOUH0085B, BF Ds 233, BF 3725 D9, BF CL05T12C13, BF J 143 4, BF 4g8B, BF 2d2A. **(B)** Distribution of CRISPR-Cas systems excluding orphan CRISPR arrays. **Type IB Type IIC Type IIIB-3 Strains:** BF S38L3, BF S38L5, BF S14. **Type IB Type IIIB-13 Strains:** BF 1007 1 F 4, BF DCMSKEJBY0001B, BF 1007 1 F 3, BF Korea 419, BF S13 L11, BF 1007 1 F 6, BF 1007 1 F 7, BF HMW 610 Bacteroides sp. UW, BF 1007 1 F 9, BF 1007 1 F 8, BF 1007 1 F 10, BF 1007 1 F 5. **Type IB Type IIC-11 Strains:** BF 3996 N B 6, BF 321, BF 1009 4 F 7, BF KLE1758, BF 3-F-2 #6, BF 322, BF 3998T B 3, BF 320, BF NCTC 9343, BF 1009 4 F 10, BF 3998 T B 4. **Type IIC Type IIIB-11 Strains:** BF 3783N1 8, BF 3783N2 1, BF 3976T7, BF DS 71, BF S23L17, BF 3783N1 2, BF S23L24, BF S23 R14, BF 3783N1 6, BF DCMOUH0042B, BF 3774 T13. **Type IB-2 Strains:** BF YCH46, BF DCMOUH0017B. **Type IIIB-29 Strains:** BF 3397 T10, BF 3986T B 10, BF DCMOUH0018B, BF 3976 T8, BF DCMOUH0067B, BF S6L8, BF J38 1, BF S36L12, BF 3986 T B 9, BF S6L3, BF DS 166, BF 3988T B 14, BF 894, BF S6R8, BF S6L5, BF S36L5, BF 3986 N B 22, BF 885, BF 3988 T1, BF 884, BF S6R6, BF 3397 N2, BF 3719A10, BF S36L11, BF 3397 N3, BF 3397 T14, BF 3986 T B 13, BF S6R5, BF 3986 N3. **Type IIC-15 Strains:** BF 34 F 2 13, BF 3719 T6, BF 3725 D9 ii, BF 2 078382 3, BF 20656 2 1, BF DS 208, BF 3986 N B 19, BF 2 F 2 4, BF 638R, BF 2 F 2 5, BF S24L26, BF 86 5443 2 2, BF S24L34, BF S24L15, BF BE1 1. **No CRISPR-Cas (with adjacent *cas* genes)-25 Strains:** BF CL05T00C42, BF BOB25, BF A7 UDC12 2, BF CL07T00C01, BF CL03T00C08, BF HMW 615, BF HMW 616, BF 2 F 2 7, BF 3 1 12, BF DCMOUH0085B, BF CL07T12C05, BF CL03T12C07, BF JCM 11017, BF Ds 233, BF JIM10, BF, BF8, BF 3725 D9, BF CL05T12C13, BF J 143 4, BF I1345, BF 4g8B, BF 2d2A, BF 20793 3, BF B1 UDC16 1, BF O: 21.

Three types of CRISPR-Cas systems (and a fourth array without associated *cas* genes) are variably distributed in *B. fragilis* strains (Table 2) and most closely match types Class 1 Type IB, Class 1 Type IIIB, and Class 2 Type IIC (Makarova et al., 2015; Figure 2); each was in a highly conserved gene neighborhood (Figure 3). One hundred strains of *B. fragilis* from those studied had one or more CRISPR-Cas systems; 9 strains had no discernable CRISPRs (Figure 4). If the Orphan CRISPR-Cas system was excluded from the analysis, 84 strains of *B. fragilis* had Type IB, Type IIIB, or Type IIC CRISPR systems. Three strains (S38L3, S38L5, and S14) harbored all three types. In most bacteria, CRISPR systems are restricted to one or possibly two types but occasional strains harboring three types of CRISPR systems were seen in *Streptococcus* and *Clostridium* species (Louwen et al., 2014). In *Streptococcus thermophiles*, for example, three different major CRISPR-Cas systems are present and function independently in crRNA biogenesis (Carte et al., 2014).

**Table 2:** Distribution of CRISPR-Cas Systems in *B. fragilis*.

**Type IB**	**Adjacent *cas* genes**	**Length^a^**	**Type IIIB**	**Adjacent *cas* genes**	**RT-*cas*^b^**	**Length^a^**	**Type IIC**	**Adjacent *cas* genes**	**Length^a^**	**Orphan**
**Consensus length DR: 29**			**Consensus length DR: 35**				**Consensus length DR: 47**			**Consensus length DR: 24**
**Average length spacer: 37**			**Average length spacer: 35**				**Average length spacer: 29**			**Average length spacer: 39**
*Bacteroides* sp. UW	IB	25	*Bacteroides* sp. UW	Alternate^f^	No	5	BF 1009 4 F 10	IIC	31,8,14	BF 1007 1 F 10
BF 1007 1 F 10	IB	15	BF 3976t7	ND^c^		5	BF 1009 4 F 7	IIC	8,7,14,31	BF 1007 1 F 3
BF 1007 1 F 3	IB	14	BF 3976t8	IIIB		5	BF 2 078382 3	IIC	19	BF 1007 1 F 4
BF 1007 1 F 4	IB	14	BF 1007 1 F 10	IIIB		15	BF 2 F 2 4	IIC	6	BF 1007 1 F 5
BF 1007 1 F 5	IB	14	BF 1007 1 F 3	IIIB		15	BF 2 F 2 5	IIC	4,5	BF 1007 1 F 6
BF 1007 1 F 6	IB	14	BF 1007 1 F 4	IIIB		15	BF 20656 2 1	IIC	15	BF 1007 1 F 7
BF 1007 1 F 7	IB	14	BF 1007 1 F 5	IIIB		15	BF 3 F 2	IIC	15,15,15	BF 1007 1 F 8
BF 1007 1 F 8	IB	6,5,(12)	BF 1007 1 F 6	IIIB		15	BF 320	IIC	5	BF 1007 1 F 9
BF 1007 1 F 9	IB	14	BF 1007 1 F 7	IIIB		15	BF 321	IIC	7,6	BF 1009 4 F 10
BF 1009 4 F 10	Truncated IB	9	BF 1007 1 F 8	ND^c^		15	BF 322	IIC	5	BF 1009 4 F 7
BF 1009 4 F 7	Truncated IB	9	BF 1007 1 F 9	IIIB		15	BF 34 F 2 13	IIC	6	BF 2 F 2 4
BF 3 F 2 #6	Truncated IB	8	BF 3397 N2	IIIB		34	BF 3719 T6	IIC	15	BF 2 F 2 5
BF 320	IB	20	BF 3397 N3	IIIB		35	BF 3725 D9 ii	IIC	32	BF 2 F 2 7
BF 321	IB	20	BF 3397 T10	ND^c^		28	BF 3774 T13	IIC	14	BF 20793 3
BF 322	IB	20	BF 3397 T14	IIIB		35	BF 3783N1 2	IIC	13	BF 3 1 12
BF 3998T B 3	Truncated IB	20,1b, inferred	BF 3719A10	IIIB		31	BF 3783N1 6	IIC	13	BF 3 F 2 #6
BF 3998 T B 4	ND^a^	3,1	BF 3774 T13	ND^c^		11	BF 3783N1 8	IIC	13	BF 3397 N2
BF DCMOUH0017B	Odd genes^d^	67	BF 3783N1 2	ND^c^		4	BF 3783N2 1	ND^a^	13	BF 3397 T14
BF DCMSKEJBY0001B	IB	25	BF 3783N1 6	IIIB		6	BF 3976T7	ND^a^	13	BF 34 F 2 13
BF HMW 610	IB	8,2^e^	BF 3783N1 8	IIIB		8	BF 3986 N B 19	IIC	14	BF 3774 T13
BF KLE1758	Truncated IB	4,2	BF 3783N2 1	ND^c^		5	BF 3996 N B 6	IIC	9,5,3	BF 3783N1 6
BF Korea 419	Truncated IB	8	BF 3986 N B 22	IIIB		7	BF 3998 T B 4	IIC	5,5	BF 3783N1 8
BF NCTC 9343	Truncated IB	8	BF 3986 N3	IIIB		15	BF 3998T B 3	IIC	5	BF 3783N2 1
BF S13 L11	ND^a^	14	BF 3986 T B 13	IIIB		7	BF 638R	IIC	29	BF 3976T7
BF S14	Truncated IB	8	BF 3986 T B 9	IIIB		7	BF 86 5443 2 2	IIC	9	BF 3986 N B 22
BF S38L5	Truncated IB	8	BF 3986T B 10	ND^c^		4	BF BE1 1	IIC	11	BF 3986 N3
BF YCH46	IB	8	BF 3988 T1	ND^c^		7	BF DCMOUH0042B	IIC	4	BF 3986 T B 13
BF s38L3	Truncated IB	8	BF 3988T B 14	ND^c^		4,24	BF DS 208	IIC	9	BF 3986 T B 9
BF 3996 NB6	IB	20	BF 884	ND^c^		8	BF DS 71	IIC	19	BF 3986T B 10
			BF 885	ND^c^		17	BF KLE1758	IIC	3	BF 3988 T1
			BF 894	IIIB		17	BF NCTC 9343	IIC	26	BF 3988T B 14
			BF DCMOUH0018B	IIIB		4,7	BF S14	IIC	24	BF A7 UDC12 2
			BF DCMOUH0042B	IIIB		17	BF S23 R14	IIC	9,10,4	BF B1 UDC16 1
			BF DCMOUH0067B	Alternate^f^	No	4	BF S23L17	IIC	14	BF BE1 1
			BF-DCMSKEJBY0001B	Alternate^f^	No	5	BF S23L24	IIC	14	BF BF8
			BF DS 166	ND^c^		12,5	BF S24L15	IIC	8	BF BOB25
			BF DS 71	ND^c^		1	BF S24L26	IIC	8	BF CL03T00C08
			BF HMW 610	Alternate^f^	No	2	BF S24L34	IIC	8	BF CL03T12C07
			BF J38 1	ND^c^		17	BF S38L3	IIC	19	BF CL07T00C01
			BF Korea 419	IIIB		15	BF S38L5	IIC	19	BF CL07T12C05
			BF S13 L11	ND^c^		5,9				BF DCMOUH0018B
			BF S14	IIIB		22				BF DCMOUH0042B
			BF S23 R14	ND^c^		21				BF DS 166
			BF S23L17	IIIB		21				BF DS 71
			BF S23L24	IIIB		21				BF HMW 616
			BF S36L11	ND^c^		24,3				BF I1345
			BF S36L12	IIIB		24,3				BF JCM 11017
			BF S36L5	IIIB		23,3				BF JIM10
			BF S38L3	IIIB		29				BF KLE1758
			BF S38L5	ND^c^		29				BF Korea 419
			BF S6L3	Alternate^f^	YES	1,3				BF NCTC 9343
			BF S6L5	ND^c^		1,3				BF O:21
			BF S6L8	ND^c^		2,4				BF S14
			BF S6R5	ND^c^		1,3				BF S23L17
			BF s6R6	IIIB		1,3				BF S23L24
			BF s6R8	ND^c^		1,3				BF S23 R14
			BF-S6L5	Alternate^f^	YES					BF S24L15
			BF-S6L8	Alternate^f^	YES					BF S24L26
			BF-S6R5	Alternate^f^	YES					BF S24L34
			BF-S6R6	Alternate^f^	YES					BF S36L11
			BF-S6R8	Alternate^f^	YES					BF S36L12
			BF-S36L11	Alternate^f^	YES					BF S36L5
			BF-S36L12	Alternate^f^	YES					BF S38L3
			BF-S36L5	Alternate^f^	YES					BF S38L5

a *Because many of the genomes are not yet assembled, the same CRISPR array may have been identified in different contigs, the length refers to the number of spacers in a particular CRISPR array*.

b *RT-cas: Gene coding for Reverse-transcriptase Cas1 fusion protein*.

c *ND: The contig on which the repeat array was found was very short and no adjacent cas genes could be identified*.

d *Has cas2, cas1, cas 4a and cas 7 only; missing the effector cas genes*.

e *BF HMW 610 has large segments of N's in the midst of what appears to be a long, continuous repeat array*.

f *Alternate gene neighborhood in the midst of polysaccharide and other metabolic genes*.

Our final data set of *B. fragilis* CRISPR-Cas arrays, reflecting manual curation, is listed in Supplementary Table 1. The CRISPR repeat arrays are written with the oldest spacer at the top of the array; therefore the adjacent *cas* genes would be located proximal to the newest spacer, at the bottom of the array. The length and sequence of repeats and the length of spacers are generally well conserved within a CRISPR locus, but may vary between CRISPRs in the same or different genomes (Rath et al., 2015); CRISPR sequences may vary in both DR and spacer sequences even among strains more than 99% at the DNA level (Kunin et al., 2007).

### Class 1 type IB CRISP-Cas systems

The closest match for the Type I Cas system is the canonic Type IB found in *Clostridium kluyveri* (Makarova et al., 2015; Figure 2A) although the gene order is different. Using the traditional annotation servers, we found genes coding for Cas2, Cas1, Cas3, Cas4a, and Cas6 (TM1814). Three additional genes, classified by all the publicly available annotation sites as hypothetical proteins, were classified as *cas5, cas7*, and *cas8b6* genes by Makarova et al. (2015) (Figure 2A); their sequences are very divergent (K. Makarova, personal communication). We did find annotated genes coding for Cas5 and Cas8 in *Bacteroides oleiciplenus* YIT 12058, but their sequences did not have any homologs in *B. fragilis* isolates.

The Type IB *cas1* gene is highly conserved among *B. fragilis* strains. It is otherwise most closely aligned with *cas1* genes from other anaerobes, including *Parabacteroides* sp., *Finegoldia magna, Clostridium tetani*, and *Clostridioides difficile*) (data not shown). The Type IB *cas3* gene is also highly conserved among *B. fragilis* strains. Its closest matches were in other *Prevotella, Porphyromonas, Chitinophaga*, and *Spirosoma* species. It was highly divergent from other *cas3* genes in the model organism GenBank databank; its closest match was with *Clostridiodes difficile* (score 126, *E*-value: 1 e-28).

Twenty-nine strains of *B. fragilis* had Type IB CRISPR-Cas systems and were found in a highly conserved gene neighborhood (Figure 3A). These CRISPR-Cas systems could be divided into two main groups. In the first group, either a full set of Type IB *cas* operon genes could be identified upstream of the CRISPR repeat, or a portion of the operon was seen but was cut off because it was at the end of the sequenced contig; in those cases, the presence of a full TYPE IB *cas* operon was inferred. The second group had CRISPR repeats with 8 or fewer spacers and only a few *cas* genes (*cas2*, truncated *cas1* (252 vs. 1014 bp), truncated *cas5* (381 vs. 564 bp) and *cas6*) were adjacent to the CRISPR repeat. The *cas4, cas7, cas8b6*, and *cas3* genes were missing in those strains, as were the 5′ and 3′ ends of *cas1* and *cas5*, respectively. The deletion of the *cas* genes, including *cas3*, and the truncation of the *cas1* and *cas5* probably occurred as a single deletion event, presumably after these spacers had been acquired, since the absence of the acquisition module probably crippled the ability of the strain to assimilate new spacer sequences. These truncated CRISPR-Cas systems, occurring in strains scattered across the dendrogram, were located in the same neighborhood as the complete Type IB CRISPR-Cas systems. All of the strains with the truncated CRISPR-Cas IB system have either Type IIIB or Type IIC systems, or both, so it is possible that the associated CRISPR-arrays could still use proteins encoded by those genes, despite their lack of the full set of Type IB *cas* genes.

Three blood isolates (*Bacteroides* sp. UW, BF DCMSKEJBY0001B and BF HMW610) had typical Type IB CRISPR-Cas systems (with adjacent *cas* genes including *cas3*) with relatively long repeat arrays (the array for HMW 610 was split into several segments because of N's in the sequencing results [data not shown]). The predicted Cas3 protein sequence of these three isolates cluster as a separate group in a phylogenetic analysis of Cas3 in *B. fragilis* as does Cas 6 and most of the other Cas proteins.

### Class 1 type IIIB CRISPR-Cas systems

Fifty-six strains had Type IIIB CRISPR-Cas systems. The Type IIIB CRISPR-Cas system had a *cas* operon upstream of the repeat segment consisting of *Cas1, CMR6, CMR5, CMR4, CMR3*, and *CMR2;* a ISNCY (i.e., Not Classified Yet) transposase was frequently located at the opposite end of these CRISPRs (Figure 2B). The *Cas1* gene in Type IIIB CRISPRs was often not annotated as *Cas1* in either RAST or NCBI annotation but as a retron-type DNA polymerase or reverse transcriptase (RT). This *cas1* gene (2271 bp) encodes a 756 aa protein that is more than twice as long as the 243 aa Cas1 protein encoded by the *cas1* gene in the Class 1 Type IB CRISPRs. These proteins contain both an RT-like superfamily domain and a Cas1_I-II-II superfamily domain (the amino acid sequence of RT-like Cas1, when present, is almost completely conserved among *B. fragilis* strains).

The RT-Cas protein is highly conserved within strains of *B. fragilis*, but very divergent from other RT-Cas 1 proteins. When compared to the landmark organisms (proteomes from 27 genomes spanning a wide taxonomic range) at NCBI, the closest matches were *Clostridiodes difficile* 630, fission yeast and *Sulfolobus acidocaldirius* (data not shown). If a wider search of the NR database was done and *Bacteroides* species eliminated, the closest matches were *Culturomica, Candidatus, Sulfuromonas*, and *Porphyromonas* species.

The *B. fragilis* Type IIIB CRISPR-Cas systems occur in highly conserved gene neighborhoods in the strains in which they are found (Figure 3B); these neighborhoods include a high percentage of genes involved in efflux processes. Frequently, a transposase gene was located just downstream of the CRISPR repeat region in those strains with adjacent Type IIIB CRISPR-Cas operons. In some cases, the contig ended just downstream of the *RT-cas1*gene; this type of sequencing result is frequently due to the presence of a transposase gene that causes breaks in the automatic scaffolding assembly of genome sequences. Transposase genes are often part of MGEs but other genes typical of mobile elements were not seen in these genomic neighborhoods. It is conceivable that the transposase gene has some function in the formation of the adjacent CRISPR (including transfer via HGT) but we could not find any mention of an association of this kind in the literature to date. The implications of the proximity of the CRISPR to the particular genomic neighborhood, if any, are unknown.

Some of the strains had Type IIIB CRISPR repeat arrays without adjacent *cas* genes in a different gene neighborhood; these included *B. fragilis* blood isolates as well as several ETBF isolates (in the ETBF isolates there were two type IIIB CRISPR arrays, one in the conserved neighborhood with adjacent *cas* genes and one in the same alternate neighborhood as the arrays in the *B. fragilis* blood isolates). These arrangements are discussed further below (virulence association with CRISPR-Cas types).

### The type IIIB *B. fragilis* CRISPR/Cas system with the Cas1-RT fusion protein are not common among bacteria

A GenBank search for proteins with both RT and Cas1 domains revealed that only 8% of bacterial Type IIIB elements have a Cas1/reverse transcriptase fusion protein, and these were most prevalent among cyanobacteria (Silas et al., 2016). The limited phylogenetic distribution of Cas1-RT and its association with only one CRISPR type suggests that there are a small number of origins of these RT-Cas1 fusions (Makarova et al., 2006; Silas et al., 2016). Recent data supports the notion that this protein may provide an efficient method to facilitate acquisition of spacers directly from RNA (Silas et al., 2016). Since other Type III CRISPR systems target RNA for degradation, these RT-associated CRISPR-Cas systems would effectively generate adaptive immunity against RNA parasites.

Further, this mechanism could target highly transcribed regions at both the DNA and RNA levels and thus serve in a regulatory capacity. The ability to target RNA substrates without targeting the bacterial chromosome would be a useful method for CRISPR regulation of endogenous genes (Sampson and Weiss, 2014). In *B. fragilis*, the conserved gene neighborhood of the Type IIIB CRISPRs included efflux operons, genes involved in cell wall biosynthesis and division, and/or genes involved in iron transport and storage.

### Class 2 type IIC CRISPR-Cas systems

Forty strains had Type IIC systems that contained the *cas2, cas1*, and *csn1 (cas9*) genes (Figure 2C). For most of the strains, there are additional ORFs between *cas1* and *cas9* that encode two hypothetical proteins annotated as putative DNA binding proteins in a cluster with Type 1 Restriction-Modification systems. These hypothetical proteins have a conserved domain annotated as RhuM; this is a group of proteins implicated in virulence/pathogenicity (RhuM, of unknown function, is encoded in the SPI-3 pathogenicity island in *Salmonella typhimurium*). Fourteen percent (46/320) of the spacers could be identified in phage sequences (Supplementary Table 2C).

Csn1 (Cas9) proteins in the Type IIC systems were highly conserved among strains of *B. fragilis* (>99% identity). The closest homologs (52–70% identity) were found in other species including *Parabacteroides*, other *Bacteroidetes, Coprobacter, Capnocytophaga*, and *Prevotella* species. Csn1 (Cas9) belongs to the COG3513 - CRISPR/Cas system Type II associated protein, and contains McrA/HNH and RuvC-like nuclease domains.

Many of the Type IIC CRISPR-Cas arrays were found on contigs that ended just downstream of the CRISPR repeat, so not much information could be gleaned about the downstream neighborhood. The upstream gene neighborhood, when it could be discerned, was highly conserved (Figure 3C).

### Short CRISPR repeats with 3 DRs and no adjacent *cas* genes (i.e., orphan CRISPRs) were found upstream of the *hipB* gene in 71 strains of *B. fragilis*

The entire sequence was completely conserved in these isolates, which originated from a variety of different sources, including normal feces, human microbiome project isolates, ETBF isolates, blood and virulent/MDR isolates. The *hipAB* operon is thought to be important in development of persister cells and multidrug tolerance in chronic infections due to *E. coli* and other bacteria (Day et al., 2004; Correia et al., 2006; Schumacher et al., 2015). HipA inhibits protein synthesis resulting in inhibition of cell growth and leading to multidrug tolerance by driving the cells into reversible dormancy and resulting in the production of persister cells (Schumacher et al., 2015). HipB binds HipA and acts as a transcriptional repressor of the *hipBA* operon (Hansen et al., 2012) and regulation of this operon is a key factor in controlling persister formation. Recently, high persister mutations were found in *E. coli* in which the mutation was not in the active site but rather interfered with higher order HipA-HipB promoter complexes that occluded the active site, thereby “unleashing” HipA to effect multidrug tolerance (Schumacher et al., 2015).

Tight regulation of *hipAB* is important since dormancy is only desirable if bacterial viability is threatened. Indeed, in global transcriptome studies of several *B. fragilis* strains we found that the *hipA* homologs were transcribed but at a very low level (manuscript submitted for publication). The ubiquitous proximity of this short orphan CRISPR to the *hipAB* operon in *B. fragilis* and the lack of adjacent *cas* genes raises the possibility that this “orphan” CRISPR is involved in regulation of *hipAB* but experimental analysis is necessary to determine whether this is the case. If confirmed, this would constitute a novel mode of regulation and could be important in the recalcitrance of chronic *B. fragilis* infections to antibiotic treatment. We did not find any significant repeat region upstream of *hipAB* in bacteria with genes phylogenetically related to *B. fragilis hipA* (*E. coli* NEB5A_07695, *E. coli* 0104: H4 str. 2011c-3493, *Shewanella oneidensis* MR-1, *Streptomyces coelicolor* and *Myxococcus xanthus*). Orphan CRISPRs (i.e., without associated *cas* genes) have been implicated in regulation in other bacteria (Sampson and Weiss, 2014). In some cases, the spacers within the CRISPR target endogenous genes and there are no associated *cas* genes. Whether the lack of adjacent *cas* genes explains why the chromosome itself is not targeted (Stern et al., 2010), or whether these CRISPRs may use CAS proteins from other locations and still be involved in regulation of the targeted genes or gene transcripts remains unclear.

### Different CRISPR/Cas subtypes have associated DR sequence subtypes and may be associated with specific functions in the cell

**Figure 5:**
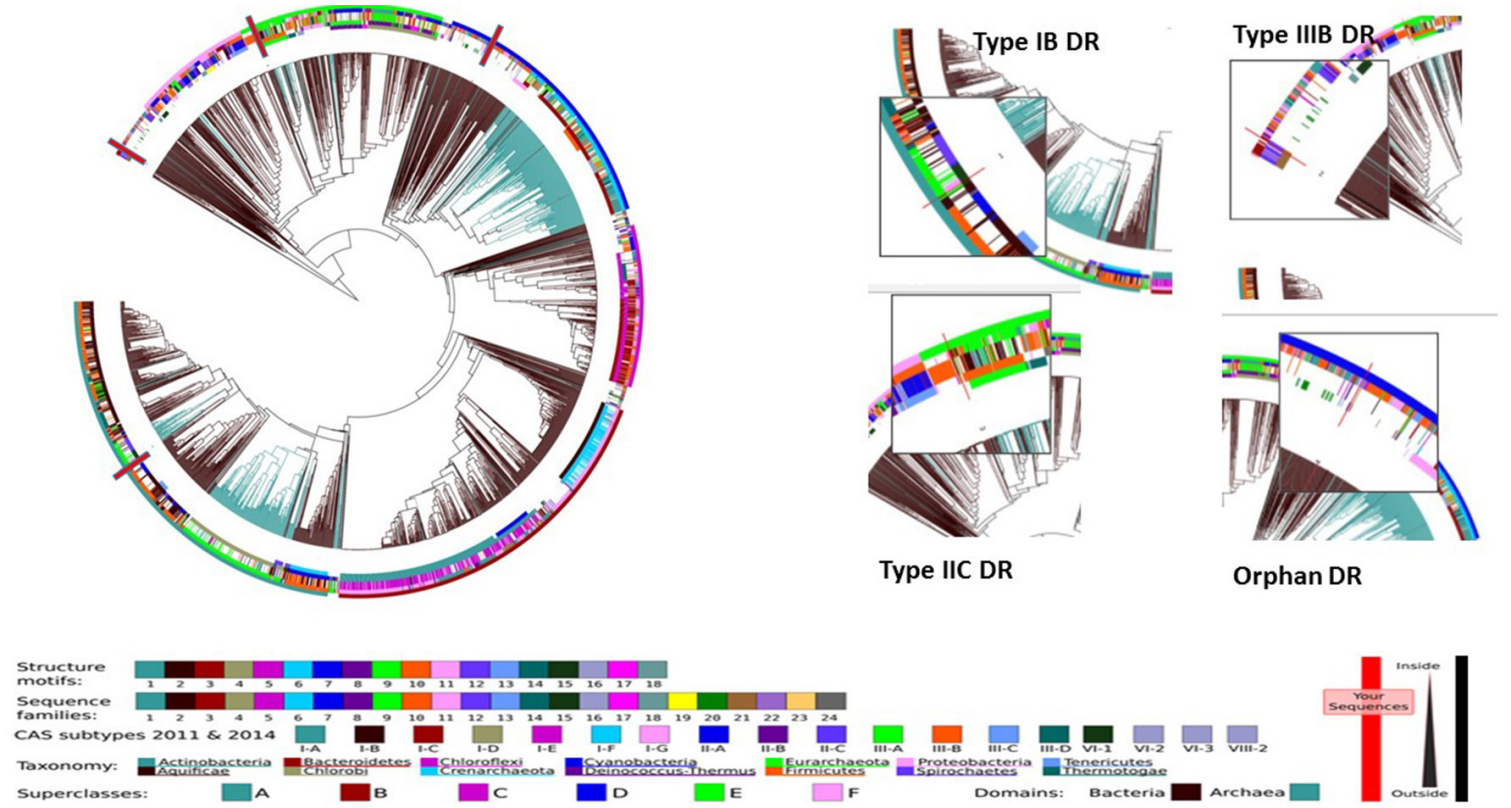
Relationship of *B. fragilis* DR (direct repeat) sequence to other sequences in the Direct Repeat Database. CRISPR Map was used to locate the consensus sequences of the four CRISPR-Cas types of *B. fragilis*. Based on placement within the map shown, Superclass, tentative taxonomy, Cas subtype and Sequence Family were determined (if available). Based on a detailed tree (not shown) of all of the DRs in the database, the closest phylogenetic neighbors were determined. **BF1_IB**: Superclass: A, Taxonomy: Bacteroidetes, Cas subtype: IB, Sequence Family: 2 (158 bacteria including 2 strains of BF). Nearest phylogenetic neighbors: *Phormidium* sp. (cyanobacteria living at temperatures of 45–60) and *Pyrococcus yayanosii* (strictly anaerobic, hypertermophilic archeon isolated from the deep sea); **BF2_IIC:** Superclass: -, Taxonomy: Bacteroidetes, Cas subtype IIC, Sequence family: 21 (23 bacteria including 2 DRs of *B. fragilis* strains). Nearest phylogenetic neighbors: *Capnocytophaga*, a gram-negative bacterium (Phylum Bacteroidetes, Family Flavobacteriaceae) normally found in the oropharangeal tract of mammals and involved in pathogenesis of animal bite wounds and periodontal disease. Remarkably, *Capnocytophaga* carries *cfxA* and *cepA*, two β-lactamase genes found in *Bacteroides* species and responsible for β-lactam resistance in *Bacteroides*. Phylogenetic analysis indicated that the Cas9 protein was also closely related to that of Cas9s found in *Capnocytophaga*; another close match was to *Fluviicola taffensis*, a novel freshwater bacterium of the family Cryomorphaceae within the phylum “Bacteroidetes”; **BF3_IIIB:** Superclass: E; Taxonomy: Firmicutes, Cas subtype IIIA? (based on arrangement of the *cas* genes, we assigned this DR to CRISPR-Cas Type IIIB). Nearest phylogenetic neighbors: *Saprospira grandis*, a gram-negative, marine, multicellular, filamentous flexibacterium, (phylum Bacteroidetes, Class: Sphingobacteria) known for devouring bacteria (and algae) and *Methanococcus vaniellii* (Superkingdom Archea, Phylum Euryarchaeota); both (particularly the latter) indicates that the CRISPR may have been horizontally transferred from a phylogenetically distant species; **BF4_Orphan** Superclass: D Taxonomy: Proteobacteria. Nearest phylogenetic neighbors: *Fluviicola taffensis*, a novel freshwater bacterium (Phylum Bacteroidetes, family Cryomorphaceae) and *Ornithobacterium rhinotracheale* (Phylum Bacteroidetes, family Flavobacteriaceae) a bacterium found worldwide that causes potentially fatal respiratory disease in poultry.

**Figure 6:**
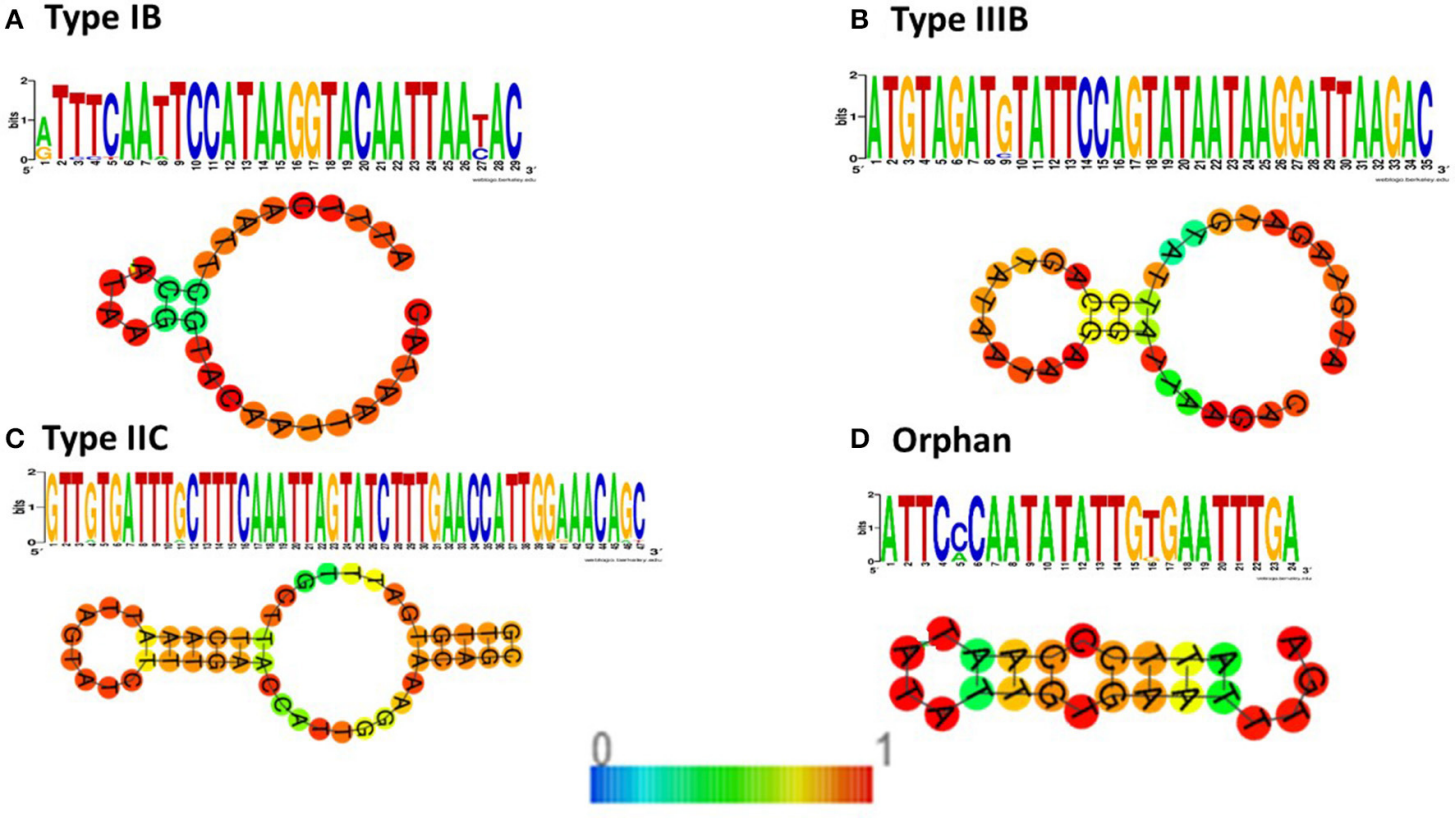
Consensus direct repeat sequences and predicted fold structure for CRISPR-Cas systems in *B. fragilis*. The structure is colored by base-pairing probabilities. For unpaired regions the color denotes the probability of being unpaired. A short bar denoting the base pairing probability is including in the drawing. **(A)** Type IB Direct Repeat. The centroid secondary structure in dot-bracket notation has a minimum free energy of 0.10 kcal/mol. **(B)** Type IIIB Direct Repeat. The centroid secondary structure in dot-bracket notation has a minimum free energy of 0.10 kcal/mol. **(C)** Type IIC Direct Repeat. The centroid secondary structure in dot-bracket notation has a very stable secondary structure with a minimum free energy of −5.30 kcal/mol. **(D)** Orphan Direct Repeat. The centroid secondary structure in dot-bracket notation has a minimum free energy of −0.90 kcal/mol.

This agrees with patterns seen in other bacteria (Kunin et al., 2007; Makarova et al., 2015) and is consistent with the notion that different sets of genes are needed to recognize, bind and process the different repeat types; the differences are probably related to the particular fold structure assumed by the DR sequence. Consensus sequences of the repeat sequence for the four CRISPR-Cas systems, their placement within the database of CRISPR direct repeat sequences, and their predicted associated RNA structure are shown in Figures 5 and 6. Figure 5 shows the placement of the four consensus repeat sequences within the entire Direct Repeat database as well as the nearest phylogenetic neighboring DRs. Notably, the four CRISPR consensus DRs were located across the entire DR spectrum, with phylogenetic neighbors from very distantly related species. More details about the phylogenetic placement of the DRs can be found in the legend to Figure 5.

The length of the consensus repeat sequence differed among the CRISPR-Cas types; Type IB (29 bp), Type IIIB (35 bp), Type IIC (47 bp), and Orphan (24 bp). This is in agreement with the finding that unusually long repeats (up to 48 bp) are exclusively present in Type IIC systems, especially in the *Bacteroidetes* phylum (Chylinski et al., 2014); the average CRISPR repeat in this system among all prokaryotes is 36 bp (Chylinski et al., 2013). The predicted fold structures of the DR sequences in each of the different CRISPR-Cas types was distinctive (Figure 6).

### Identification of protospacers and spacer distribution within each of the CRISPR types are shown in Table 2

The search itself is somewhat problematic because the spacer sequences are so short. We used both the CRISPR Target program and NCBI BLAST with specified parameters to search for protospacer matches. Occasionally, a particular gene was identified to which the spacer had significant homology but, more often, other CRISPR arrays were identified. We repeated the search using the NCBI BLAST engine but adding a filter to screen out Repeat Arrays; we were able to identify protospacer matches for some additional spacer sequences but more often the match was to non-related nucleotide sequences.

We found that 7% of the spacer sequences were significantly homologous to a *Bacteroides* phage sequence. The majority of protospacers could not be assigned to a specific sequence (phage, plasmid or other bacterial gene or even inter-gene sequences) and most were identified in CRISPR regions in other *B. fragilis* strains. These numbers are consistent with other studies of prokaryotic CRISPR-Cas arrays; for example, in *Riemerella* strains, only 13/153 (8%) of spacers were homologous with a phage or plasmid target (Zhu et al., 2016).

In closely related strains, spacer identity and placement was frequently highly conserved. Spacers common to multiple strains can be due to either clonal dispersion of the CRISPR element through heredity or to HGT of these CRISPRs. The identified spacers, along with the strains in which they are found, their placement in those strains, and their predicted protospacer target are listed in Supplementary Tables 2A–C. A visual representation of their distribution is shown in Supplementary Tables 3A–C (binary distribution tables in Excel format).

One hundred thirty three unique spacers were found in the Type IB CRISPR-Cas systems. Five of the spacer targets matched, imperfectly but across the entire spacer length, to a phage sequence; these spacers were all found in an atypical, extremely long (67 spacers!) CRISPR repeat array in the blood isolate BF DCMOUH0017B. Notably, this array had an incomplete set of adjacent *cas* genes (only *cas1, cas2*, and *cas4*) and two transposases (one IS21 type) immediately upstream of *cas1*. Cas1, Cas2, and often Cas4 comprise the adaptation module that is responsible for new spacer insertion (Makarova et al., 2015). The very long length of this CRISPR repeat array and multiple spacers matching *B. fragilis* phage protospacers is consistent with a CRISPR-Cas system that can acquire new spacers but cannot defend against the invading nucleic acid.

Type IIC systems contained significantly higher portion of spacers with crRNA homologous to a phage sequence than were found in the other systems (87% of spacers matching a phage sequence belonged to Type IIC CRISPR-Cas systems, 13% belonged to Type IB systems).

Spacer distribution in the Type IIIB CRISPR-Cas systems is shown in Supplementary Table 2B and graphically in Supplementary Table 3B. There were 252 unique spacers in the Type IIIB system, and the distribution was very broad. Strains with close phylogenetic relationship had identical spacers (e.g., BF 1007 F-2 thru F-10; BF 320, BF 321, and BF 322). Fifteen of the spacers (15/252, 6%) had matches with prokaryotic genes; no matches with phage or plasmid genes were identified and most matches were with other CRISPR regions (Supplementary Table 2B).

Type III CRISPR-Cas systems are associated with degradation of phage DNA in *Staphylococcus epidermidis* (Jiang et al., 2016). However, in *Streptococcus pyogenes*, there is a strong bias of the Type II system for acquisition of spacers matching viral protospacers (Heler et al., 2015). In our study, as in others, the DR repeat structures and associated *cas* genes of the two types were very distinct. Apparently, more than one CRISPR-Cas systems with the associated DR repeat structure can be used for acquisition and/or degradation of phage genetic material.

### Leader sequences in CRISPR-Cas systems

A 193 bp leader sequence was identified in the Type IB system of BF YCH 46 using CRISPRleader version 3.0 (Alkhnbashi et al., 2016) (kindly analyzed for us by Dr. Omer Alkhanbashi). The sequence was located between the repeat array and the *cas* genes and was extremely conserved among the Type IB CRISPR-Cas systems identified in *B. fragilis*. (Leader sequence: (5′-TGTTATTGTGAATTATCAATGGTAAAGTAACGGAAACGCTCTATGACATGTTGATATATAGATGTTTAGTACCTATGTCGCTAACCTATGTTTTTTATATTATTCTTGATCGACACATTATTTCTAAAGAAAAGCAATTTTCTGCAAAAGCATTTGGCTTATTACTAAGTAATTGCGTTGATTGATGGGTAGA-3′). Strains BF HMW610, *Bacteroides* sp. UW, BF DCMSKEJBY0001B and BF DCMOUH0017B, all phylogenetically related BF blood isolates, had slightly divergent Type IB leader sequences (data not shown). The promoter sequences for *B. fragilis* are not completely established and no obvious promoter sequence was found on the leader sequence (Bayley et al., 2000). While no specific leader sequence could be definitively assigned in the Type IIIB or Type IIC systems using the CRISPRleader program, the sequences upstream of the CRISPR repeat array (and downstream of the *cas* operon) were highly conserved, respectively, in each of those two CRISPR-Cas types. As was found for the Type IB leader, strains BF HMW610, *Bacteroides* sp. UW, BF DCMSKEJBY0001B and BF DCMOUH0017B had slightly divergent leader sequences for both Types IIIB and IIC systems.

### No “typical” CRISPR systems could be identified on mobile elements

Phylogenetic analysis indicates that CRISPR-Cas arrays have undergone extensive HGT, possibly on megaplasmids, as very similar *cas* genes are found in distantly related organisms (Godde and Bickerton, 2006). CRISPR-Cas arrays were found on a variety of MGEs (Sorek et al., 2008) including *Clostridium butyricum* megaplasmids (Iacobino et al., 2013) and viruses attacking *Cyanobacter* (Chenard et al., 2016). We examined a variety of mobile elements in *B. fragilis* for the presence of CRISPR arrays. Ten plasmids and/or conjugative transposons for which there is partial or complete sequence were examined by CRT and did not have any discernable CRISPRs. These included: pBF9343, CTnHybL, pHAG88, p610 88, BOB_25 PAO, CTn86, CTn341, CTnDot, and CTnPg1-a (note: many of the conjugative transposons in *B. fragilis* were only partially sequenced). Additionally, no CRISPRs were detected in the large horizontally transferred chromosomal fragments recently described (Husain et al., 2017). We also examined available sequences for 14 *B. fragilis* plasmids and did not find any CRISPR elements contained within their sequences. The examined plasmids (and their Genbank accession numbers) were: 2-078382-3, pBFP53, complete sequence, (CM004523.1); DCMOUH0042B, pBFU42e contig 1, (JPGQ01000001.1); pBFU42e, insertion sequence ISBf13 (complete), (KJ417513.1); pBF69566b, (KJ830768.1); pBF69566a, (KJ830769.1); 20656-2-1, pBI143, (LIDU01000064.1); IS4351, R plasmid encoding macrolide B resistance, (M17124.1); JIM 10, unnamed plasmid, (MBRB01000037.1); JIM 10, unnamed plasmid, (NZ_CM004507.1) pBFP35, complete sequence, (NC_011073.1); pBFUK1, complete sequence, (NC_019534.1);; 2-078382-3, pBFP53, (NZ_CM004523.1); DCMOUH0042B, pBFU42e contig 1, (NZ_JPGQ01000001.1); pBI143, complete sequence, (U30316.1). No CRISPRs could be identified on the two sequenced BF phages (B40-8 and B124-14). However, using the more relaxed parameters in the CRISPR finder program, a potential CRISPR repeat was identified in pBF9343 but was not associated with adjacent *cas* genes. It should be noted that many of the CRISPRs that were identified on the genome had adjacent genes (e.g., transposase, mobility *(mob)* genes) that are typically found in mobile elements, so it is certainly possible that these CRISPRs are MGE-associated but that the exact MGE is not defined.

### Transposase genes were frequently found adjacent to CRISPR systems

Since the CAS proteins themselves possess transposase activity, it is not clear why it would be advantageous to have another transposase gene in close proximity. The presence of these genes, which are ubiquitous on mobile elements, might suggest that these CRISPRs are (or were in the past) contained within a putative MGE. There are ancestral innate immune systems that were formed from transposon-like elements containing *cas1* and *cas2*, eventually using the terminal inverted repeats characteristic of the transposon to form the ancestral CRISPR repeats that were then duplicated by *cas1* (*cas1* also functioned in addition of spacers) (Nuñez et al., 2015). The molecular mechanism of CRISPR spacer integration is similar to that of both retroviral integration and DNA transposition that are mediated by integrases/transposases (Rath et al., 2015). Thus, it is possible that this adjacent transposase has some sort of function with the CRISPR function itself; however, that is purely speculative.

### Association of virulence in *B. fragilis* strains with specific CRISPR-Cas systems

The presence of CRISPR-Cas systems are variably associated with virulence (Makarova et al., 2011; Barrangou, 2015) and/or antibiotic resistance in pathogenic bacteria. On the one hand, the ability of a bacterium to incorporate mobile elements bearing pathogenic determinants would argue for a less robust CRISPR-Cas defense system; on the other hand, the presence of multiple CRISPRs systems with a variety of spacers may indicate previous exposure to these elements, parts of which were incorporated into the bacterial genome.

The *B. fragilis* strains isolated from blood (Table 1) that clustered into a tight phylogenetic group (Figure 1) had distinctive properties in their CRISPR-Cas content. None of these strains, with the exception of BF DCMOUH0042B (which clearly has a different lineage) had CRISPR-Cas systems belonging to the Type IIC CRISPR-Cas system. The distinct lack of the Type IIC CRISPR-Cas system for the majority of the blood isolates might suggest that the ability to incorporate phage DNA (which often carries antimicrobial resistance or virulence genes) is a beneficial trait for these virulent strains.

Further, the while these isolates did contain Type IIIB CRISPR repeat arrays, they differed from the typical Type IIIB array in two ways: (1) they were located in a completely different gene neighborhood (Figure 3B) and (2) had no adjacent *cas* genes. Interestingly, several of the ETBF isolates contained two sets of Type IIIB CRISPR-Cas repeat arrays: one in the typical Type IIIB gene neighborhood with a full set of adjacent Type IIIB *cas* genes and one (without adjacent *cas* genes) in the same neighborhood as the Type IIIB blood isolate CRISPR arrays.

No significant target sequences for the blood isolate spacers were identified, except for one spacer in BF DCMOUH0018B with significant homology to a region carrying the *B. fragilis* insertion sequence IS1168 and *nimB* (nitroimidazole resistance) gene. The particular spacers carried by these isolates, despite the lack of Type IIIB *cas* genes, could be due either to them acquiring them at some point via an intact Type IIIB CRISPR-Cas system that they subsequently lost (perhaps during the transfer to an alternate gene neighborhood) or by HGT of the repeat array (without the *cas* genes) from another strain that did contain those spacers.

**Figure 7:**
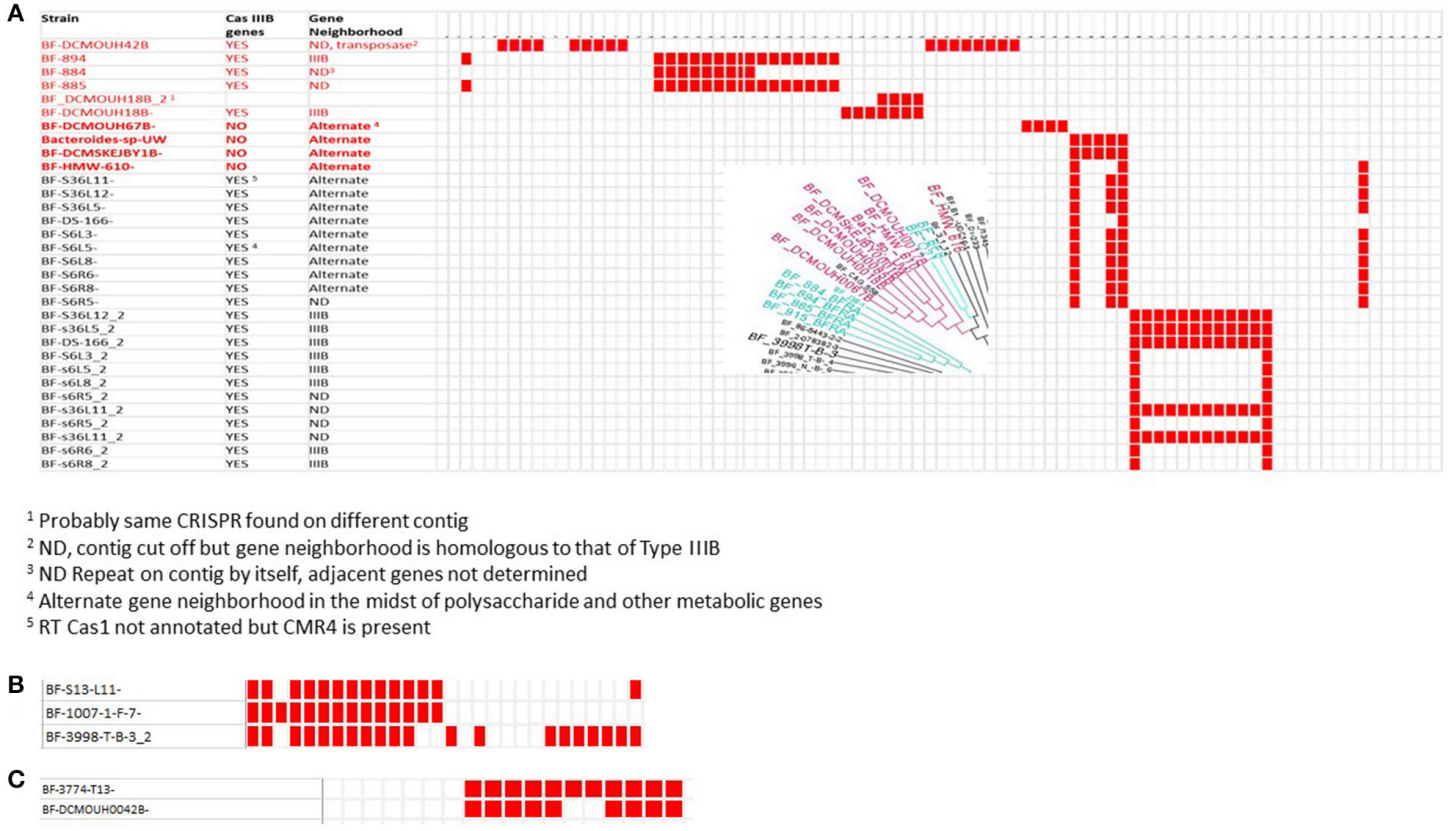
Binary representation of spacer distribution in Type IIIB CRISPR-Cas in blood isolates and other strains with repeat arrays in alternate neighborhoods. The CRISPRs are configured with the oldest spacer at the “top” of the array (i.e., spacer 1, to the left edge of the binary representation) thus the newest spacers are those at the right edge. **(A)** This is an excerpted panel of the full binary representation of spacer distribution in Supplementary Table 3B. Each unique spacer is represented by a red vertical bar. Isolates from blood are in a red font. A unique pattern of spacers are found in the atypical neighborhood. Four of the blood isolates have no other Type IIIB CRISPR-Cas array and no Type IIIB *cas* genes. There are no *cas* genes adjacent to the CRISPR array in the atypical gene neighborhood, but since there is also a Type IIIB CRISPR-Cas array in the typical neighborhood, it is possible that the genes can act *in trans* on the atypical array. This is not the case for the four blood isolates (in bold red font). Blood isolates *Bacteroides* sp. UW and BF-DCMSKEJBY18 have two additional spacers not seen in other isolates but their protospacer targets could not be determined. A cutout of the dendrogram shown in Figure 1 is superimposed to show the phylogenetic relationship of the blood isolates. **(B)** Type IB spacer distribution in three strains of BF. BF-S13-L11 and BF-1007_1_F-7 are closely related phylogenetically while BF-3998_T-B-3_2 is at the opposite end of the phylogenetic tree; the more ancient part of the CRISPR (i.e., the first spacers) is highly conserved. BF-3998-T-B-3_2 has a longer array with more spacers at the newest edge, indicating that BF-3998-T-B-3_2 acquired more spacers. **(C)** Type IIIB spacer arrangements in two closely related strains of BF (see Figure 1). This pattern is consistent with the frequently seen homology of CRISPR arrays between highly related strains, with one strain losing two of the internal spacers. Another possible but less likely scenario is that BF-3774_T13 picked up two additional spacers that were not added at the leading edge but at an internal location.

Figure 7A is an excerpted panel of the full binary representation of spacer distribution in Supplementary Table 3B and highlights the spacer distribution of the atypical Type IIIB CRISPR-Cas arrays in blood isolates (red font) as well as in those isolates that have both typical and atypical Type IIIB systems. Each unique spacer is represented by a red vertical bar. Four of the blood isolates have no other Type IIIB CRISPR-Cas array and no Type IIIB *cas* genes anywhere on the genome. Some of the ETBF isolates also have an array containing these same spacers, and also in the same “atypical” neighborhood. But these isolates *also* have another Type IIIB array, in the traditional neighborhood with adjacent *cas* genes; thus, it is possible that the genes can act *in trans* on the atypical array. Again, this is not the case for isolates *Bacteroides* sp. UW, BF-DCMSKEJBY18, BF HMW 610, and BF-DCMOUH0067B. Blood isolates *Bacteroides* sp. UW and BF-DCMSKEJBY18 have two additional spacers not seen in other isolates. It is not clear how these atypical systems appeared but it is tempting to speculate that the atypical array broke away from the original CRISPR-Cas array and that the very virulent blood isolates somehow discarded the *cas* genes portion of the original array. At this point we have no evidence for that speculation nor a reason that it would be beneficial for those isolates to discard that array or adjacent genes. Figures 7B and C illustrate examples of spacer pattern diversions between closely related strains and spacer conservation between distant strains. Figure 7B shows distribution of two closely related strains and one distant strain and Figure 7C shows Type IIIB spacer arrangements in two closely related strains of BF and is consistent with a pattern in which spacers can get lost.

### The plasticity of the *B. fragilis* genome is balanced by multiple systems to avoid invading DNA elements

The *B. fragilis* genome is very “plastic,” due both to its ability to incorporate pathogenicity islands from other *B. fragilis* and foreign genes via HGT as well as its ability to simply turn specific genes on or off as needed. Combined, these traits allow *B. fragilis* to adapt to new nutrition pathways, utilize specific efflux pumps to rid the cell of toxic substrates, and display new surface epitopes—taken together, allowing them to change from friendly commensal to dangerous threat (Wexler, 2007). We previously demonstrated transfer of a mobile element that contained multiple resistance genes of aerobic origin clustered within a conjugative transposon (Husain et al., 2014).

This ability of *B. fragilis* to easily incorporate foreign genes is intriguing since *B. fragilis* also possesses strong DNA restriction modification (DNA/RM) systems to degrade “non-self” DNA. We previously demonstrated horizontal transfer of mobile elements bearing alternate variants of these genes from a multidrug resistant *B. fragilis* isolate (HMW 615) to *B. fragilis* 638R (Husain et al., 2017). These systems are located in shufflons with invertible promoters that can be turned off or on, so that the bacterium can control whether the incoming DNA will be degraded (Patrick et al., 2010). If the system is turned “off,” the incoming DNA can survive degradation. External or internal signals that regulate these systems have not yet been described. Now we have shown that in addition to the DNA/RM systems, there are abundant CRISPR elements in *B. fragilis* with adjacent *cas* genes, suggesting that these CRISPRs are indeed active as a bacterial defense system. We had previously suggested that predation by bacteriophages is an evolutionary driving force for generation of variable polysaccharide and R-M systems in *B. fragilis* (Patrick et al., 2010) and that this system can be diversified by HGT (Husain et al., 2017). In an interesting twist, *B. fragilis* also has a Type IIC CRISPR system with a high proportion of crRNAs with homology to *B. fragilis* phages; it is likely that this CRISPR system is largely directed to protect against invading bacteriophage (but these systems are lacking in the most virulent *B. fragilis* strains).

Thus, at least two unique systems are in place in *B. fragilis* to control and degrade incoming DNA, despite the well-established role of *B. fragilis* as a “resistance reservoir” (Salyers et al., 2004; Coyne et al., 2014) and its known ability to incorporate both other *B. fragilis* elements as well as “foreign” genes (Husain et al., 2014). It seems likely that an intricate balance of these two evolutionary forces is responsible for the remarkable adaptability of *B. fragilis* to the changing nutritional availability, immune forces and competitive organisms in the complex environment of the gut microbiome.

## Conclusions

In this study, we identified and described CRISPR-Cas systems in *B. fragilis* as a first step for further exploration of the roles and mode of function of *B. fragilis* CRISPRs. We now need to determine whether the *B. fragilis* CRISPRs are functional in bacterial immunity and/or in regulation and whether they can signal acquisition of virulence determinants. Aside from these associations, there is growing evidence that CRISPR-Cas systems may have alternative roles that allow the bacterium to survive host defenses and replicate (Sampson and Weiss, 2014). For example, the Cas1 protein of the CRISPR-Cas system of *E. coli* may play a role in DNA repair (Babu et al., 2011). Since DNA damage to bacteria can be the result of specific host defenses during infection (e.g., the production of radical nitrogen and oxygen species), it would be beneficial to the bacterium to possess redundancy in its DNA repair capability (Sampson and Weiss, 2014). An additional way to avoid or repair DNA damage due to radical oxygen species would obviously be of great benefit to the anaerobic *B. fragilis* as well. In addition to these functions, there are reports of CRISPR-Cas involvement in resistance to stress, pathogenicity and regulation of biofilm formation (Barrangou, 2015).

We found that the most virulent strains of *B. fragilis*, the blood isolates, did not have Type IIC CRISPR-Cas systems, which may suggest that they remain capable of incorporating resistance and virulence factors that may be transferred via phages. Also, they do not appear to have functional Type IIIB systems, although they retain the CRISPR repeat array characteristic of that system, but in a distinct gene neighborhood. Thus, it appears that for these most virulent blood isolates, there is a benefit to being able to acquire genes from phages and possibly other mobile elements, which is completely consistent with the evidence that many antimicrobial resistance genes and other virulence genes are mobilized via HGT. Further analysis of the spacer acquisition pattern will help to determine how these spacers were acquired and help to elucidate the extent to which strains evolve by vertical evolution and/or horizontal gene transmission. Experimental molecular manipulation to determine the functions of the various CRISPR arrays will lead to a more comprehensive understanding of their functions in bacterial defense, gene regulation, virulence and commensalism of this important gut pathobiont.

## Author contributions

MT contributed to the design, performed bioinformatic analyses, assisted in the interpretation of results and in writing the manuscript. HW designed the study, interpreted the results and wrote the manuscript. All authors have read and approved the final version of the manuscript.

### Conflict of interest statement

The authors declare that the research was conducted in the absence of any commercial or financial relationships that could be construed as a potential conflict of interest. The reviewer AO and handling Editor declared their shared affiliation.
